# Distinct patterns of vital sign and inflammatory marker responses in adults with suspected bloodstream infection

**DOI:** 10.1016/j.jinf.2024.106156

**Published:** 2024-05

**Authors:** Qingze Gu, Jia Wei, Chang Ho Yoon, Kevin Yuan, Nicola Jones, Andrew Brent, Martin Llewelyn, Tim E.A. Peto, Koen B. Pouwels, David W. Eyre, A.Sarah Walker

**Affiliations:** a Nuffield Department of Medicine, University of Oxford, Oxford, UK; b Big Data Institute, Nuffield Department of Population Health, University of Oxford, Oxford, UK; c Oxford University Hospitals NHS Foundation Trust, Oxford, UK; d Health Economics Research Centre, Nuffield Department of Population Health, University of Oxford, Oxford, UK; e Brighton and Sussex Medical School, Brighton, UK; f NIHR Health Protection Research Unit in Healthcare Associated Infections and Antimicrobial Resistance, University of Oxford, Oxford, UK

**Keywords:** Bloodstream infection, C-reactive protein, Vital signs, Trajectory subgroups, Centile chart

## Abstract

**Objectives:**

To identify patterns in inflammatory marker and vital sign responses in adult with suspected bloodstream infection (BSI) and define expected trends in normal recovery.

**Methods:**

We included patients ≥16 y from Oxford University Hospitals with a blood culture taken between 1-January-2016 and 28-June-2021. We used linear and latent class mixed models to estimate trajectories in C-reactive protein (CRP), white blood count, heart rate, respiratory rate and temperature and identify CRP response subgroups. Centile charts for expected CRP responses were constructed via the lambda-mu-sigma method.

**Results:**

In 88,348 suspected BSI episodes; 6908 (7.8%) were culture-positive with a probable pathogen, 4309 (4.9%) contained potential contaminants, and 77,131(87.3%) were culture-negative. CRP levels generally peaked 1–2 days after blood culture collection, with varying responses for different pathogens and infection sources (p < 0.0001). We identified five CRP trajectory subgroups: peak on day 1 (36,091; 46.3%) or 2 (4529; 5.8%), slow recovery (10,666; 13.7%), peak on day 6 (743; 1.0%), and low response (25,928; 33.3%). Centile reference charts tracking normal responses were constructed from those peaking on day 1/2.

**Conclusions:**

CRP and other infection response markers rise and recover differently depending on clinical syndrome and pathogen involved. However, centile reference charts, that account for these differences, can be used to track if patients are recovering as expected and to help personalise infection treatment.

## Highlights

• We investigated vital sign/laboratory test trajectories in >75,000 suspected bloodstream infections.
• Responses differed across specific clinical syndromes and pathogens.
• Distinct patterns in CRP included normal, slow and delayed/limited recoveries.
• We developed CRP centile charts characterising typical responses to guide individualised management.

## Introduction

Timely initiation of active antimicrobials is an important priority in sepsis,^1^ but blood cultures identify a pathogen in only up to 30–40% of bloodstream infections (BSI).^1^, ^2^ Hence, most antimicrobials are started, and often continued, empirically. In this context, key questions include “how do I know my patient is responding to treatment?” and “when should I switch antibiotics?” as potentially not providing adequate treatment in some patients leads to more severe infections and higher mortality.^3^ Conversely, over use of empirical broad-spectrum antibiotics, either initially or as escalation for inadequate response contributes risks increasing antimicrobial resistance and hence worse outcomes over the longer term.^4^

Where blood cultures are negative or results not yet available, laboratory test results (e.g. C-reactive protein (CRP),^5^, ^6^, ^7^, ^8^, ^9^, ^10^ procalcitonin^2^, ^6^, ^9^, ^10^, ^11^ and white blood cell (WBC) counts^11^) and vital signs (e.g. temperature, heart rate and respiratory rate^12^) can guide treatment decisions. Scoring systems, including the Sequential Organ Failure Assessment (SOFA) Score, can also provide insights into status and risk of deterioration.^1^ Among these, the role of serial CRP measurements in monitoring treatment response has been previously studied.^13^, ^14^, ^15^, ^16^, ^17^ Patterns of relative CRP change (i.e., ratio of measurements on day 4 or 5 to those at baseline) has been found to correlate with clinical outcomes.^13^, ^16^, ^18^ However, host responses are heterogeneous, both at presentation and throughout infections,^12^, ^19^ due to factors such as the causative pathogens, sources of infection, and individual patient characteristics. The relationship between routinely collected clinical parameters and these factors has not been well described.

Detailed electronic health records (EHRs) potentially allow identification of different patient response trajectories and underlying heterogeneity. Additionally, reference “normal” clinical responses given a patient’s status at presentation and effective treatment could be constructed using centile-based methods, as in paediatric growth charts,^20^ and used to identify deviations from typical recovery to inform individualised clinical decision-making. Compared to calculating CRP ratios, this could provide a more dynamic and visually intuitive means of assessing treatment response and is applicable to various clinical parameters. Previous studies used group-based models to identify subgroups of patients with different vital signs, WBC and SOFA score trajectories in patients with suspected sepsis^12^, ^21^, ^22^, ^23^, ^24^, ^25^, ^26^, ^27^, ^28^; however, to date, none have applied centile-based methods to infection responses.

We therefore aimed: first, to estimate changes in routinely collected clinical parameters over suspected BSI episodes (defined by negative or positive blood cultures, stratified by pathogen/clinical syndrome; second, to identify different response patterns using latent class trajectory modelling, to identify those responding standardly to (effective) antimicrobials and other treatment; and third, to construct centile reference charts for expected clinical response in standard responders to support clinicians tailoring treatment to individual patient responses.

## Methods

We used de-identified data from the Infections in Oxfordshire Research Database, containing information from all inpatient admissions at the Oxford University Hospitals NHS Foundation Trust (OUH), United Kingdom, together with vital signs, microbiology and biochemistry/haematology results and antimicrobials prescribed in hospital. OUH contains ∼1100 beds in four hospitals, providing all acute care and pathology services to a population of ∼750,000 and specialist services to the surrounding region. Ethical approval was obtained from the National Research Ethics Service South Central Oxford C Research Ethics Committee (19/SC/0403) and the National Confidentiality Advisory Group (19/CAG/0144).

We included suspected BSI episodes occurring in adults (≥16 years old) defined by blood being collected for culture during a hospital admission, as a marker of suspected bloodstream infection. There was no excluding based on comorbidity or other factors, as we aimed to capture a broad range of patients with suspected BSI (Fig. S1). A new suspected BSI episode was defined if there were >14 days since the last blood culture. Where more than one set of blood cultures were obtained within a potential BSI episode, we prioritised pathogens, then contaminants, then any negative cultures as the index blood culture defining each episode’s start (date/time of the blood collection for culture). Episodes with index blood cultures taken >24 h before admission or after discharge were excluded; those taken in the 24 h prior to admission were included to capture blood cultures taken in the Emergency Department.

### Statistical analyses

To estimate trajectories of CRP, WBC and vital signs (heart rate, respiratory rate, tympanic temperature) over the course of an infection episode we used linear mixed models, including all measurements from −1 day (CRP/WBC) or −6 h (vital signs) before to +8 days after the start of each episode. We incorporated non-linearity in these trajectories over time using natural cubic splines (as fixed and random (episode-specific) effects), and adjusted (as fixed effects) for infection source (identified from antimicrobial prescribing indications^29^), community-onset (≤48 h after admission), blood culture result (positive, potential contaminant, negative) and pathogen group (based on genus and clinical significance, Table S1), age, sex, Charlson and Elixhauser scores and immunosuppression, and any interactions with time if interaction-p < 0.05. Separate adjusted models were fitted to examine effects of infection source (not adjusting for pathogen group which would not be known at presentation) and baseline antimicrobial susceptibility (determined by microbiological tests and intrinsic resistance^30^) (see Supplement for details of all statistical analyses).

To identify and classify into groups the variation (population-level heterogeneity) in CRP response trajectories, we used unadjusted latent class mixed models^31^ (details in Supplement), assigning each episode to the class (group) with highest posterior probability. Classes exhibiting a rapid rise and prompt fall were assumed to represent patients responding standardly to (assumed effective) antibiotics and other treatments. We used this approach rather than trying to adjust for post-baseline changes in antimicrobials because of potential time-dependent confounding. It also allowed us to include culture-negative episodes; this is important because hospital-level empirical antibiotic recommendations are based on susceptibility data from recent previous infections, with treatment switched promptly if a resistant pathogen is identified in an individual patient. However, many infections are culture-negative, so resistant infections may be missed, and identifying culture-positive resistant infections may take several days. To compare 30-day all-cause mortality between different latent classes we used multivariable logistic regression.

Centile reference charts (analogous to paediatric growth charts) for expected CRP responses in standard responders with peak response on day 1/2 were constructed using the lambda-mu-sigma method^32^ and bootstrapping, assuming that the observed episodes’ characteristics would generalise to the population presenting to hospital with suspected BSI (see Supplement).

## Results

From 1-January-2016 to 28-June-2021, 24.4% (95,928/392,443) admissions had blood cultures taken (39.5% [82,535/208,699] emergency and 4.4% [7132/163,201] elective admissions; overall 122 blood cultures/1000 patient-days). After deduplication of blood cultures taken within 14 days, there were 88,348 suspected BSI episodes in 60,647 patients (Fig. S1); a single Gram-positive pathogen was identified in 1912 (2.2%), a single Gram-negative pathogen in 3736 (4.2%), 1260 (1.4%) had other pathogens or were polymicrobial, 4309 (4.9%) had only a potential contaminant, and 77,131 (87.3%) were culture-negative (Table 1). The median age was 67.3 (IQR 48.5–80.4) years. Patients had relatively few comorbidities (median Charlson 1 (IQR 0–2)), with only 12,802 (14.5%) episodes in immunosuppressed patients; most episodes were community-onset (71,258, 80.7%). Respiratory (22,818; 25.8%), multiple (11,012; 12.5%) or urinary (9275; 10.5%) sources were most common. Only 6728 (7.6%) had undergone a surgical procedure in the 14 days prior to their index blood culture.

**Table 1:** Characteristics at the start of 88,348 suspected bloodstream infection (BSI) episodes between 01-January-2016 and 28-June-2021. Percentages in the header are of all episodes, and in the main body are column percentages within each group; continuous variables are summarised using median (IQR). Baseline NEWS score was calculated using the closest set of vital signs within 1 day before to 1 day after the start of each episode.

Characteristic	Gram-positive pathogens N = 1912 (2.2%)^a^	Gram-negative pathogens N = 3736 (4.2%)^a^	Other pathogens N = 1260 (1.4%)^a^	Potential contaminant (s) N = 4309 (4.9%)^a^	Culture-negative N = 77,131 (87.3%)^a^	Overall N = 88,348 (100%)^a^
Age at admission (years)	70.6 (54.4, 82.1)	74.9 (61.7, 84.1)	65.3 (48.5, 78.9)	69.0 (52.8, 81.2)	66.6 (47.4, 80.1)	67.3 (48.5, 80.4)
Sex (male)	1144 (59.8%)	2080 (55.7%)	691 (54.8%)	2215 (51.4%)	37,558 (48.7%)	43,688 (49.4%)
Ethnicity						
White	1589 (83.1%)	3044 (81.5%)	980 (77.8%)	3449 (80.0%)	61,647 (79.9%)	70,709 (80.0%)
Other	64 (3.3%)	178 (4.8%)	70 (5.6%)	261 (6.1%)	4631 (6.0%)	5204 (5.9%)
Unknown	259 (13.5%)	514 (13.8%)	210 (16.7%)	599 (13.9%)	10,853 (14.1%)	12,435 (14.1%)
Charlson score	1 (1, 2)	2 (1, 3)	1 (0, 2)	1 (0, 3)	1 (0, 2)	1 (0, 2)
Elixhauser score	3 (2, 5)	3 (2, 5)	3 (1, 4)	3 (1, 4)	2 (1, 4)	2 (1, 4)
NEWS score (baseline)	4 (2, 6)	4 (2, 6)	3 (2, 6)	3 (1, 5)	2 (1, 4)	3 (1, 5)
Unknown	131 (6.9%)	210 (5.6%)	117 (9.3%)	442 (10.3%)	6684 (8.7%)	7584 (8.6%)
Immunosuppression	310 (16.2%)	719 (19.2%)	306 (24.3%)	662 (15.4%)	10,805 (14.0%)	12,802 (14.5%)
Diabetes mellitus	490 (25.6%)	972 (26.0%)	254 (20.2%)	971 (22.5%)	14,415 (18.7%)	17,102 (19.4%)
Palliative care	179 (9.4%)	359 (9.6%)	144 (11.4%)	308 (7.1%)	3986 (5.2%)	4976 (5.6%)
Community-onset	1505 (78.7%)	2852 (76.3%)	863 (68.5%)	3074 (71.3%)	62,964 (81.6%)	71,258 (80.7%)
Source of infection						
Respiratory	431 (22.5%)	378 (10.1%)	185 (14.7%)	1180 (27.4%)	20,644 (26.8%)	22,818 (25.8%)
Multiple sources	421 (22.0%)	817 (21.9%)	228 (18.1%)	566 (13.1%)	8980 (11.6%)	11,012 (12.5%)
Urinary	132 (6.9%)	1044 (27.9%)	116 (9.2%)	427 (9.9%)	7556 (9.8%)	9275 (10.5%)
Abdominal	101 (5.3%)	590 (15.8%)	169 (13.4%)	254 (5.9%)	5798 (7.5%)	6912 (7.8%)
Skin, soft tissue, orthopaedic	272 (14.2%)	88 (2.4%)	80 (6.3%)	261 (6.1%)	5596 (7.3%)	6297 (7.1%)
CNS	35 (1.8%)	23 (0.6%)	21 (1.7%)	72 (1.7%)	1063 (1.4%)	1214 (1.4%)
Other	117 (6.1%)	60 (1.6%)	87 (6.9%)	160 (3.7%)	2432 (3.2%)	2856 (3.2%)
Unspecific	403 (21.1%)	736 (19.7%)	374 (29.7%)	1389 (32.2%)	25,062 (32.5%)	27,964 (31.7%)

a Median (IQR); n (%).

### CRP response trajectories following negative/positive blood cultures

**Fig. 1:**
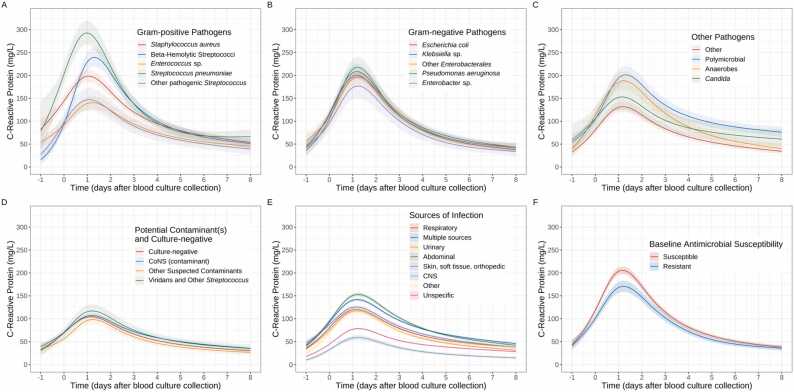
CRP response trajectories following different blood culture results (Gram-positive pathogens (A), Gram-negative pathogens (B), other pathogens (C), and potential contaminants and culture-negative results (D); adjusted for source of infection and other covariates), sources of infection (E) (not adjusted for blood culture results but adjusted for other covariates) and baseline antimicrobial susceptibilities (F) (adjusted for blood culture results, source of infection and other covariates). See Fig. S2A for response trajectories of no baseline antimicrobial recorded and unknown baseline susceptibility. Predictions are plotted at the reference values of other adjusting variables: age = 64 years, male, Charlson score = 1, Elixhauser score = 3, community-onset, absence of immunosuppression, urinary source (excluding panel E), and *E. coli* infection (panel F only). Nonlinear trends were incorporated via natural cubic splines with four knots at the 20th, 40th, 60th and 80th percentiles of observed time values (day 0, day 0.8, day 2.4, day 4.7).

Seventy-seven thousand nine hundred and fifty-seven (88.2%) suspected BSI episodes in 54,381 (89.7%) patients had ≥1 CRP available (median 4 (IQR 2–6, range 1–20) measurements/episode), these were broadly similar to episodes without CRP measurements (standardised mean difference (SMD) ≤ 0.12, Table S1) with the exception of slightly fewer culture-negative results in those with CRP results (SMD = 0.22). CRP increased sharply, generally peaking between day 1 and 2 post blood-culture collection, with varying rates of increase and peaks for different pathogen groups (interaction p < 0.0001, Fig. 1). Adjusted CRP response trajectories differed most substantially in Gram-positive infections (Fig. 1A), rising much faster and earlier with *Streptococcus pneumoniae* infections than other Gram-positive (or Gram-negative) pathogens and peaking at day 1 (mean level ∼290 mg/L), followed by rapid declines and near stability by day 6 (∼65 mg/L). CRP also increased rapidly with beta-haemolytic Streptococci but peaked slightly later, reaching ∼240 mg/L on day 1.3 and then decreasing rapidly (to ∼50 mg/L by day 8). CRP response trajectories for Gram-negative infections were broadly similar to each other, peaking at 175–215 mg/L after day-1 before falling back to ∼35 mg/L (Fig. 1B). For other pathogens, peak CRP levels were higher in episodes with anaerobic and polymicrobial infections (190–200 mg/L), and the latter had the slowest recovery rate, remaining at ∼75 mg/L by day 8; recovery was also slower in *Candida* bloodstream infection episodes (∼60 mg/L by day 8, Fig. 1C). CRP responses were still seen in those with only potential contaminants or no organism identified, and were similar to each other, peaking at lower mean levels (95–115 mg/L, at just after 24 h) to those with pathogens identified (Fig. 1D).

We considered infection source in separate adjusted models not including pathogen group (since most blood cultures were negative, and clinical syndromes but not blood culture results were known at admission). Differences between most sources were smaller than between pathogen groups (Fig. 1E). Episodes with abdominal or multiple source(s) elicited the strongest CRP responses, with mean levels reaching ∼150 mg/L and ∼130 mg/L respectively by day 1. CRP responses were lower for episodes with neurological and non-specific origin, with peaks of ∼60 mg/L and ∼80 mg/L. There was little difference between the remaining sources. In culture-positive infections, adjusting for pathogen group and infection source, episodes with pathogens susceptible to any baseline antimicrobial elicited higher CRP responses than those resistant to all baseline antimicrobials (∼205 mg/L vs. ∼170 mg/L on day 1.2, Fig. 1F; Fig. S2; Table S2).

Additionally, after adjusting for infection source and pathogen group, CRP levels were independently higher in males (∼20 mg/L higher peak vs. females, Fig. S3A), in episodes with nosocomial onset (20–60 mg/L higher during the episode vs. community-onset, time-interaction p < 0.0001, Fig. S3B) and immunosuppressed patients after day 3 (time-interaction p < 0.0001, Fig. S3C), older patients up to 70 years (∼9 mg/L higher per 10 years older, Fig. S3D) and those with lower Charlson comorbidity scores (Fig. S3E).

### Response trajectories for other physiological measurements

Similar adjusted associations were observed for other physiological measurements, although to a lesser extent than for CRP (Figs. S4–S7). WBC peaked earlier than CRP, whereas heart rate, respiratory rate and temperature all declined rapidly over the first day: however, differences associated with different pathogen groups were consistent. Specifically infections with *S. pneumoniae* and beta-haemolytic Streptococci had the highest initial heart rate, respiratory rate and temperature, at ∼105 beats/minute, 22–23 breaths/minute and 37.9–38.2°C 6 h before blood culture collection, dropping to ∼83 beats/minute, ∼18 breaths/minute and ∼36.7°C by day 2 (Figs. S4A/S5A/S6A) and the highest WBC count, peaking around the time of blood culture collection at ∼16 × 10^9^/L (Fig. S7A). Similar to CRP, recovery was slower in patients with *Candida* and polymicrobial infections (Figs. S4C/S5C/S6C/S7C), but response trajectories for other pathogen groups and sources of infection (Figs. S4E/S5E/S6E/S7E) were broadly similar to each other. There was little difference in response trajectories for other physiological measurements between susceptible and resistant baseline pathogens (Fig. S4F/S5F/S6F/S7F).

### Underlying heterogeneity in CRP response trajectories

**Fig. 2:**
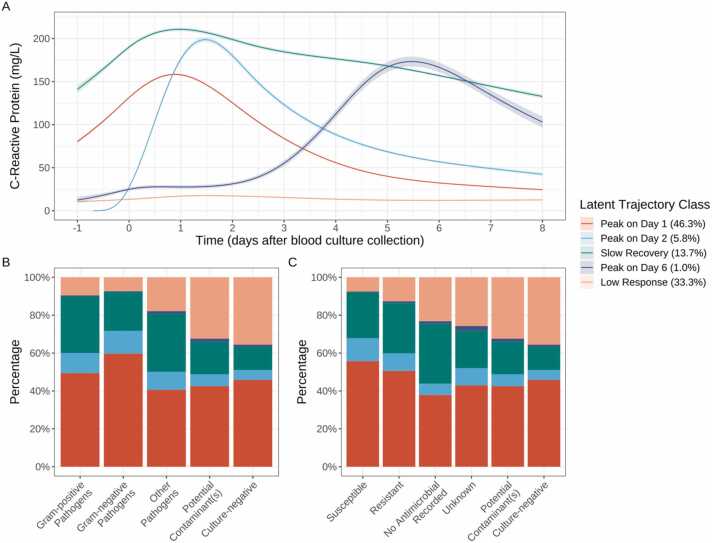
Latent classes of CRP response trajectories (A) (unadjusted for other covariates), distribution of the latent trajectory classes by pathogens identified (B) and baseline antimicrobial susceptibility (C). See Figs. S9A and S9D for the distribution of blood culture results and baseline antimicrobial susceptibilities across each latent trajectory group.

Latent class modelling identified five different underlying CRP response subgroups (Fig. 2A, Table 2, Fig. S8), distinguished by having their peak on day 1 (36,091 [46.3%]) or day 2 (4529 [5.8%]), slow recovery (10,666 [13.7%]), peak on day 6 (743 [1.0%]) and low values throughout (25,928 [33.3%]). Overall, 34,466 (51.1%) culture-negative and 1917 (48.8%) contaminant-only episodes still had acute CRP responses (peaking day 1/2) followed by typical recovery vs. 4237 (64.5%) pathogen-positive episodes (Fig. 2B). For pathogen-positive episodes with susceptibility results, 67.8% (3401/5017) with susceptible baseline antimicrobials had peak CRP on day 1/2, vs. 59.8% (529/884) with resistant baseline antimicrobials (Fig. 2C).

**Table 2:** Characteristics of 77,957 suspected BSI episodes (with ≥1 measurement of CRP within 1 day before to 8 days after the start of each episode) by predicted latent trajectory class. See Table S1 for comparison of pathogens isolated from included vs. excluded episodes. The percentages in the header are of all episodes included, and in the main body are column percentages out of the total number of episodes within each distinct latent class; continuous variables are summarised using median (IQR). See Supplement for definition of baseline antimicrobial susceptibility.

Characteristic	Peak on day 1, N = 36,091 (46.3%)^a^	Peak on day 2, N = 4529 (5.8%)^a^	Slow recovery, N = 10,666 (13.7%)^a^	Peak on day 6, N = 743 (1.0%)^a^	Low response, N = 25,928 (33.3%)^a^	Overall, N = 77 957 (100%)^a^
Class-membership probability	0.6 (0.5, 0.7)	0.9 (0.6, 1.0)	0.7 (0.5, 0.9)	0.8 (0.6, 1.0)	0.7 (0.6, 0.9)	0.6 (0.5, 0.8)
Age at admission (years)	69.2 (51.6, 81.1)	68.4 (46.7, 81.7)	70.3 (56.4, 81.0)	70.1 (56.0, 81.6)	63.6 (43.6, 79.3)	67.8 (49.6, 80.6)
Sex (male)	18,865 (52.3%)	2240 (49.5%)	6178 (57.9%)	415 (55.9%)	11,363 (43.8%)	39,061 (50.1%)
Charlson score	1 (0, 2)	1 (0, 2)	1 (1, 3)	2 (1, 3)	1 (0, 2)	1 (0, 2)
Elixhauser score	2 (1, 4)	2 (1, 4)	3 (2, 4)	3 (2, 4)	2 (1, 4)	2 (1, 4)
Community-onset	28,184 (78.1%)	4010 (88.5%)	7162 (67.1%)	521 (70.1%)	22,189 (85.6%)	62,066 (79.6%)
Immunosuppression	4909 (13.6%)	533 (11.8%)	2222 (20.8%)	164 (22.1%)	3797 (14.6%)	11,625 (14.9%)
Diabetes mellitus	7225 (20.0%)	921 (20.3%)	2335 (21.9%)	158 (21.3%)	4862 (18.8%)	15,501 (19.9%)
Palliative care	1995 (5.5%)	200 (4.4%)	1332 (12.5%)	79 (10.6%)	892 (3.4%)	4498 (5.8%)
>1 blood cultures in episode	9386 (26.0%)	1792 (39.6%)	6096 (57.2%)	495 (66.6%)	4688 (18.1%)	22,457 (28.8%)
>1 positive blood cultures in episode	706 (2.0%)	136 (3.0%)	663 (6.2%)	27 (3.6%)	191 (0.7%)	1723 (2.2%)
Baseline antimicrobial susceptibility						
Culture-negative	30,916 (85.7%)	3550 (78.4%)	8352 (78.3%)	642 (86.4%)	24,004 (92.6%)	67,464 (86.5%)
Potential contaminant(s)	1672 (4.6%)	245 (5.4%)	673 (6.3%)	65 (8.7%)	1274 (4.9%)	3929 (5.0%)
Susceptible	2795 (7.7%)	606 (13.4%)	1220 (11.4%)	17 (2.3%)	379 (1.5%)	5017 (6.4%)
Resistant	447 (1.2%)	82 (1.8%)	235 (2.2%)	8 (1.1%)	112 (0.4%)	884 (1.1%)
No antimicrobial recorded	176 (0.5%)	28 (0.6%)	147 (1.4%)	6 (0.8%)	108 (0.4%)	465 (0.6%)
Unknown	85 (0.2%)	18 (0.4%)	39 (0.4%)	5 (0.7%)	51 (0.2%)	198 (0.3%)

a Median (IQR); n (%).

In subgroups with peaks on day 1/2, CRP levels initially rose dramatically, then dropped and stabilised by day 8 (Fig. 2A). The subgroup peaking on day 2, however, had lower starting levels, potentially due to enrichment with community-onset infections (88.5% vs. 78.1%, SMD = 0.28, Table S3). More of those peaking on day 2 also had >1 blood culture in their episode (39.6% vs. 26.0%, SMD = 0.29, Table S3), and more had pathogens cultured (16.2% [734/4529] vs. 9.7% [3503/36,091], Fig. S9A). The slow recovery subgroup had the highest peak yet recovered the slowest. Compared with those peaking on day 1, this subgroup were older (median 70.3 vs. 69.2 years, SMD = 0.13), had more comorbidities, immunosuppression (20.8% vs. 13.6%, SMD = 0.19), nosocomial infections (32.9% vs. 21.9%, SMD = 0.25), >1 positive blood cultures in the episode (6.2% vs. 2.0%, SMD = 0.22) and more resistance to baseline antimicrobials (2.2% vs. 1.2%, SMD = 0.2, Table S3). The very small subgroup who peaked ∼6 days had a similar profile to the slow recovery subgroup, with even more comorbidities and episodes with >1 blood culture (66.6% vs. 26.0% in those peaking on day 1, SMD = 0.89, Table S3). Mean CRP in the low response subgroup remained <20 mg/L throughout; this subgroup had fewer repeat blood cultures (18.1% with >1 blood culture vs. 26.0% in those peaking on day 1, SMD = 0.19), more negative cultures (92.6% vs. 85.7%, SMD = 0.32) and more community-onset suspected infections (85.6% vs. 78.1%, SMD = 0.20); patients were also generally younger (median 63.6 vs. 69.2 years, SMD = 0.19, Table S3).

After adjusting for potential confounders, compared with the day 1 peak subgroup, 30-day all-cause mortality (which occurred in 8386 (9.5%) suspected BSI episodes) was significantly higher in the slow recovery subgroup (odds ratio (OR) = 2.00 [95%CI 1.85, 2.17], p < 0.001) and the day 6 peak subgroup (OR = 1.95 [1.50, 2.51], p < 0.001) (Table S4), and significantly lower in the low (CRP) response subgroup (OR = 0.76 [0.71, 0.83], p < 0.001). There was no evidence of a difference in 30-day all-cause mortality between those peaking on day 2 and day 1 (OR = 0.89 [0.78, 1.03], p = 0.12).

Estimated response trajectories for heart rate, respiratory rate, temperature and WBC count by the latent CRP trajectory class showed the same response subgroup patterns in terms of early/delayed/low response (Fig. S10).

### Expected CRP response

**Fig. 3:**
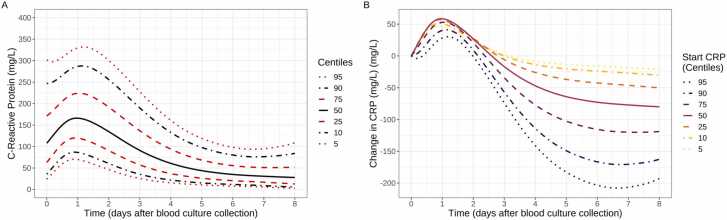
Centile reference chart of expected CRP response in patients with culture-positive/negative suspected BSI responding standardly to antimicrobials (A) and change in CRP from initial value in centile (B). Change in CRP was calculated by subtracting the CRP value at the datetime of blood culture collection. Note: estimated from the two latent classes peaking on days 1 and 2 in Fig. 2, regardless of pathogen isolated.

**Fig. 4:**
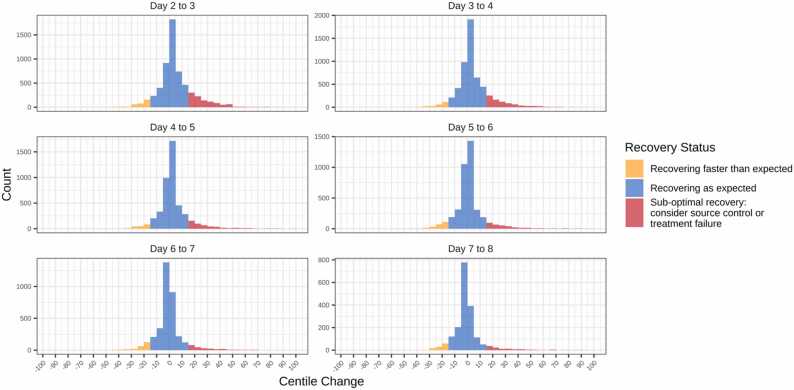
Distribution of centile changes between two consecutive days. Centile change <−15, −15 to 15 and >15 were considered as recovering faster than expected (orange), recovering as expected (blue) and sub-optimal recovery (red), respectively. Calculations were based on 13,635 episodes (5548 peak on D1, 1168 peak on D2, 4443 slow recovery, 359 peak on D6 and 2117 low response) that had CRP measurements on two consecutive days (26,019 pairs of CRP measurements) and focused on the recovery stage, starting from day 2 onwards, to allow for initial measurements to be influenced by time since presentation.

We included the 40,620 episodes in the subgroups with CRP peaking on day 1 or day 2, i.e., the response that would be clinically expected to an infectious insult followed by an uncomplicated recovery, in order to estimate “normal” response to suspected BSI treated with effective antimicrobials (either empirically or through prompt switching), and expected underlying variation, regardless of whether a pathogen was identified. Estimated centile charts based on 100,000 bootstrap samples show 5th, 10th, 25th, 50th, 75th, 90th, and 95th percentiles of a normal CRP response to suspected BSI, whether subsequently culture-positive or not (Fig. 3A) Estimates were similar randomly sampling one measurement per patient, suggesting limited potential bias from multiple measurements per episode (Fig. S11). Overall, median CRP peaked at ∼165 mg/L on day 1 (24 h after blood culture collection), then decreasing gradually to ∼25 mg/L by day 8. This chart clearly illustrates the challenges on relying on absolute CRP value to determine response, or even change in CRP (Fig. 3B), given individual-level heterogeneity. For example, a value of 150 mg/L would be expected (50th percentile) 12 h after blood culture collection for an average responder, but would still represent a standard (i.e. good) response at 2.7 days for a patient whose initial CRP was higher (75th percentile) and even later, at 3.7 and 4.2 days, for even higher initial CRP (90th and 95th percentile respectively). Expected CRP response centiles (10th, 50th, 90th) estimated separately for different infection sources showed little difference to the overall centiles (Fig. S12). Our analysis of individual patients' CRP changes against the population-level centile showed that most patients tended to track along the centile curves during the recovery stage (Fig. 4, see Supplement for details).

## Discussion

Using large-scale EHR data, we show clinical response trajectories in laboratory tests and vital signs are associated with both specific blood culture results and infection sources in patients with suspected BSI. We found considerable variation across different pathogen groups in response trajectories, with much of the variation seen in differences around presentation. Five distinct patterns of CRP response trajectories were identified using latent class models, providing evidence for heterogeneity in infection responses; interestingly nearly 65% of culture-positive episodes with a pathogen, but also around half of culture-negative episodes, were associated with acute responses. Centile reference charts were created based on the “typical” CRP responders to standardise assessment of infection progression and treatment response in patients with suspected BSI; these could be used to guide management independent of microbiological test results.

In response to an inflammatory stimulus, such as an infection, CRP levels usually begin to rise within 6–12 h, peak around 36–50 h, and then decline with a half-life of approximately 19 h once the stimulus is removed.^33^, ^34^, ^35^ Our findings are generally consistent with these known dynamics, as we observed CRP levels peaking around day 1–2 post-blood culture collection in most subgroups. However, we have shown that response trajectories were strongly associated with infection sources and culture results/pathogen groups, at least partly explaining heterogeneity in host responses to BSI. Higher and more prolonged inflammatory responses with abdominal and multiple sources of infection are consistent with earlier studies,^36^, ^37^ and associations with higher mortality.^36^ Previous evidence on associations between pathogen groups and biomarker responses is inconsistent. Several studies have reported stronger inflammatory responses (e.g., in procalcitonin and CRP) in patients with Gram-negative infections,^37^, ^38^, ^39^ however, similar to our findings, others have found higher CRP levels in patients with Gram-positive infections, especially by *S. pneumoniae*.^40^, ^41^ This inconsistency may be caused by differences in the timing of single-point measurements or variability in patient cohorts with restricted sample size (174−5267). Based on serial measurements from a broader population with suspected BSI, we found more pronounced CRP, WBC, heart rate, respiratory rate, and temperature responses to some Gram-positive bacteria (particularly *S. pneumoniae* and beta-haemolytic Streptococci) after adjusting for infection sources. Infections susceptible to baseline antimicrobials were associated with higher CRP responses than resistant infections, possibly due to increased initial inflammatory responses from antimicrobial killing or reduced fitness costs from antimicrobial resistance. However, this difference was not apparent in other physiological measurements.

We considered those in the subgroups with peak CRP levels on day 1 or day 2 (52.1% episodes) to have a “normal” response, also representing appropriate antimicrobial treatment in those with bacterial infection. The day 2 peak subgroup may represent a slightly delayed response or detection of suspected BSI earlier in the illness. The slow recovery subgroup was characterised by stronger initial and more persistently elevated CRP and, like the small subgroup with a delayed peak on day 6, included more older patients with more comorbidities and had higher 30-day all-cause mortality rates compared with those peaking on day 1. The slow recovery subgroup had more repeated positive blood cultures, suggesting persistent infection due to either lack of source control or treatment failure. A modestly higher proportion of episodes with inactive initial antimicrobial therapy in this subgroup may suggest that antimicrobial resistance could be a contributing factor to persistent infection in some cases. However, our study does not provide definitive evidence to differentiate between source control failure and treatment failure, as the underlying reasons for persistent infection were not systematically recorded. The subgroup with limited CRP response included younger patients with more negative blood cultures, with mean estimates likely reduced by the absence of bacterial infection or a systemic response in a substantial subset.

Whilst host response characteristics and clinical outcomes have previously been used to sub-phenotype patients with suspected BSI or sepsis, CRP response trajectories have not been described in this detail to our knowledge. Several studies defined 3 to 4 CRP ratio response patterns based on changes in follow-up CRP relative to baseline CRP values,^13^, ^15^, ^16^, ^18^ and relatively consistent response patterns were observed in serially measured body temperatures and SOFA scores, but not in WBC.^16^ We identified five distinct response subgroups based on CRP response trajectories and observed consistent response patterns in heart rate, respiratory rate, body temperature, and white blood cell counts. Others have applied similar approaches to longitudinal vital signs, WBC, or SOFA score.^12^, ^22^, ^23^, ^24^, ^25^, ^26^, ^27^, ^28^ Broadly mirroring our observations, three studies identified four temperature trajectory groups using measurements within the first 72 h: "hyperthermic, slow resolvers”, "hyperthermic, fast resolvers”, “normothermic”, and “hypothermic”.^21^, ^22^, ^23^ Our subgroup with CRP peaking on day 1/2 had temperature responses corresponding to the "hyperthermic, fast resolvers”; our late CRP response subgroup likely corresponded to the “hypothermic” group, both comprising older patients with more comorbidities. However, although our slow recovery CRP subgroup had a similar temperature trajectory to the “hyperthermic, slow resolvers”, our subgroup consisted mainly of relatively old rather than young patients as previously. WBC response trajectories estimated by our latent CRP subgroups were also broadly consistent with a previous study identifying seven WBC trajectories from 917 ICU patients.^25^

Despite the heterogeneity in CRP responses by pathogens and clinical syndromes, for a given initial CRP value, responses were relatively consistent, meaning they could be summarised using a single centile reference chart. This heterogeneity in CRP response trajectories illustrates the limitations of a “one-size-fits-all” approach to using absolute CRP values, or even change in CRP, to determine escalation, de-escalation or duration of antimicrobial therapy in patients with suspected BSI (since absolute values/changes mean something different depending on initial CRP values), whereas the centile chart provides a potentially useful alternative. Spotting unexpected deterioration and biomarker-guided antibiotic stewardship are key potential applications.^5^, ^42^ Although previous biomarker-guided stewardship reduced antibiotic prescription and duration while demonstrating non-inferior or lower mortality, compliance remained suboptimal.^43^, ^44^, ^45^, ^46^ The centile reference chart provides a more visually intuitive means of assessing response, potentially aiding clinical decisions by incorporating individual-level observations alongside evidence-based references. Its implementation could be supported by embedding it within EHR systems.

Study strengths include our large sample size (77,957 suspected BSI episodes) and longer follow-up (8 days) compared to previous studies, and using comprehensive clinical data over several years. However, one limitation is that the clinical measures we considered can be elevated for several reasons: we therefore used latent class models to identify subgroups with responses typical of an effectively treated infection. We only adjusted for a pre-specified set of potential confounders given the complexity of the linear mixed models: the effects of other potential confounders will have been captured within the episode-level random effects. Data were collected for clinical reasons, and CRP and other laboratory measurements are less likely to be (serially) repeated in those making a good recovery; measurements at later time-points are therefore likely enriched for elevated values. Hence true expected trajectories may fall more rapidly and more completely than we estimate. Mitigating this entirely would require sampling irrespective of clinical progress and post-discharge, unlikely feasible at scale. Similarly, due to the lack of data on the timing of blood culture collection relative to the onset of symptoms in EHRs, biomarker trajectories were estimated relative to the first blood culture collection which may differ from onset of symptoms, potentially explaining the two subgroups with CRP peaking on day 1 or 2. However, the relationship between onset of symptoms and the initial inflammatory insult is also unknown, and symptom onset may be subjectively reported. Future studies could aim to collect more detailed information on symptom onset and duration to better characterise the temporal dynamics of host response in suspected BSI. Additionally, the use of alternative statistical methods, such as alignment algorithms or time-warping techniques, could be explored to account for variations in the timing of clinical measurements relative to disease onset. It is also possible that some patients within the “normal response” group had elevated CRP measurements for other reasons in the absence of infections, including trauma, recent surgery, and inflammatory conditions such as pancreatitis; although these are likely to account for a minority of episodes. It is also an intriguing possibility that similar CRP centile charts could be used to determine normal post-operative recovery patterns, with deviations potentially indicative of surgical site infection.

Other limitations include the absence of CRP measurements in 10,391 (11.8%) episodes. Our relatively high culture-negative rate (87.3%) is partly due to our broad definition of suspected infection and historically high rate of taking blood cultures; nevertheless 51.1% culture-negative episodes still exhibited typical CRP responses, with peaks on day 1/2, suggesting that many of these episodes likely represent true infections. The inclusion of these culture-negative episodes in our analysis helps to provide a more comprehensive and generalisable understanding of biomarker response patterns in suspected BSI. Only the association between baseline antimicrobial activity and CRP response was examined; future planned work includes investigating associations between CRP levels/centiles and changes in antimicrobial therapy, both to assess if there is evidence that sub-optimal CRP responses lead to changes in antimicrobials and also if switching from inactive to active therapy changes CRP trajectories. Procalcitonin can also help guide antimicrobial therapy duration, but this biomarker was not measured routinely at our hospitals. While our study focussed on single marker trajectories, we acknowledge the potential of integrating multiple biomarkers into a composite panel; our results may inform subsequent investigations in this area. Despite using bootstrapping and simulations, EHR data may contain inaccuracies or missing information, potentially impacting the estimation of clinical response trajectories. Furthermore, our analysis was limited to patient data available in one, albeit large, hospital group, which might influence generalisability.

In summary, we found strong associations between clinical response trajectories and both infection sources and different pathogen groups in patients with suspected BSI, with distinct CRP response patterns, reflecting normal, slow, and delayed or limited responses. Considering the dynamic nature of BSI and sepsis and heterogeneity in individual CRP response trajectories, the centile reference charts developed here may provide a valuable tool for guiding individualised infection management. Future research should focus on exploring the dynamic association between response and antimicrobial use and evaluating the practical application of centile reference charts in clinical settings.

## Acknowledgements and funding

This work was supported by the National Institute for Health Research Health Protection Research Unit (NIHR HPRU) in Healthcare Associated Infections and Antimicrobial Resistance at 10.13039/501100000769Oxford University in partnership with the UK Health Security Agency (NIHR200915), and the NIHR Biomedical Research Centre, Oxford. DWE is a Big Data Institute Robertson Fellow. ASW is an NIHR Senior Investigator. The views expressed are those of the authors and not necessarily those of the NHS, the NIHR, the Department of Health or the UK Health Security Agency. The funders had no role in study design, data collection and analysis, decision to publish, or preparation of the manuscript.

## Declaration of Competing Interest

The authors declare that they have no known competing financial interests or personal relationships that could have appeared to influence the work reported in this paper.

## Data Availability

The data analysed are available from the Infections in Oxfordshire Research Database (https://oxfordbrc.nihr.ac.uk/research-themes/modernising-medical-microbiology-and-big-infection-diagnostics/iord-about/), subject to an application and research proposal meeting on the ethical and governance requirements of the Database.
