# Supplementary material for: Distinct patterns of vital sign and inflammatory marker responses in adults with suspected bloodstream infection
Source: J Infect. 2024 May;88(5):None. doi: 10.1016/j.jinf.2024.106156 (PMC11893474; doi:10.1016/j.jinf.2024.106156)
Supplement: Supplementary file 1 — Supplementary material [file mmc1.docx]

# Tracking normal inflammatory marker and vital sign responses in adults with suspected bloodstream infection: Supplementary materials

# Supplementary Methods

The aim of this analysis was to use routinely collected electronic health records to estimate trajectories of routinely collected parameters (CRP, WBC and vital signs (heart rate, respiratory rate, tympanic temperature)) over the course of an infection episode, investigate underlying heterogeneity in responses and how these related to specific patient factors and assess whether centile reference charts for expected clinical response could be a helpful tool for clinicians, for example to tailor treatment. Routine electronic health record data were available for blood taken for cultures (including organism and susceptibility results), demographics, inpatient admissions (dates and times, specialty, associated diagnostic codes), laboratory tests (CRP, WBC), vital signs, antimicrobial prescriptions (date/time of administration and indication (free text)).

## Population

We defined a new suspected bloodstream infection (BSI) episode when there was >14 days since the last collection of blood for culture using the culture dataset containing pseudonymised patient identifier, date and time of blood sample collection, and blood culture results (including antimicrobial susceptibilities). The index blood culture in each infection episode was determined by a hierarchy prioritising pathogen presence over contaminants and negative results. We then merged demographic data, and excluded episodes in patients <16 years at the index blood culture and a small number of episodes with missing demographic information (**Figure S1**). After merging with information about inpatient admissions (including dates and diagnostic codes) we lastly excluded infection episodes with index blood cultures drawn 24 hours prior to inpatient admission or after discharge (**Figure S1**).

We chose to use the time of collection of blood for the index culture as time zero in analyses because this provided a consistent reference point across all episodes, and for potential subsequent changes in management. This may be considered a proxy for the clinical recognition of a potential infection. However, it is later than the onset of symptoms and the initial infectious insult, and does not account for variations in these versus blood collection. Assessing symptom onset is inevitably subjective and this information is also not available in routine healthcare data so could not be used in this analysis.

## Outcomes

All CRP, WBC and vital sign measurements within relevant time windows from the index blood culture were extracted based on pseudonymised patient identifiers (total 213,632 CRP measurements, 232,021 WBC, 1,774,103 heart rate, 1,751,985 respiratory rate, 1,760,520 temperature). Episodes without CRP measurements were excluded from the trajectory analysis (**Figure S1**).

## Covariates

We defined community-onset infections as those where the index blood culture was taken ≤48h after admission. Charlson comorbidity and Elixhauser acuity scores were calculated using ICD-10 diagnostic codes based on a 1-year lookback period, adding all primary and secondary ICD-10 codes in the year prior to the inpatient episode containing the index blood culture to all secondary ICD-10 codes from the current inpatient episode^1^. Immunosuppression was determined by the presence of ICD-10 diagnostic codes for AIDS/HIV, metastatic cancer, haematological malignancies, primary immunodeficiencies and end-stage liver disease within the same 1-year lookback period. Similarly, palliative care was determined by the presence of ICD-10 diagnostic codes for palliative care within the same 1-year lookback period. Infection sources were identified from free text antimicrobial prescribing indications within 1 day before to 8 days after the start of each episode using a pre-developed natural language processing model^2^.

Baseline antimicrobials were defined as those administered within 12h before to 24h after index blood culture collection. The baseline antimicrobial susceptibility profile for each suspected BSI episode was determined from results of antimicrobial susceptibility testing as well as information on intrinsic resistance and antimicrobial activity in the Sanford Guide if susceptibility test results were not available for specific drugs (including both antibiotics and antifungals)^3^. Where results were not available and there was uncertainty about the expected susceptibility then results were recorded as unknown. The susceptibility profile of rarer pathogens was defined as unknown where these were not tested and intrinsic susceptibility data were not available. Episodes where the patient was admitted directly to the intensive care unit, where antimicrobial administration was recorded using a different EHR system from which data were not available (31 cases) were also defined as having unknown susceptibility. For monomicrobial infections, episodes were defined as susceptible if any antimicrobial received during the baseline window was susceptible to the pathogen isolated (including single dose gentamicin), otherwise as resistant if the pathogen eventually isolated was resistant to all antimicrobials received in the baseline window, otherwise as unknown. For polymicrobial infections, resistance of any one pathogen to all baseline antimicrobials defined the episode as resistant. Candida infections treated with only baseline antibiotics (79.0%[64/81]) were also defined as resistant to baseline therapy.

## Statistical models

Linear mixed models were used to estimate CRP, WBC and vital signs’ trajectories throughout suspected BSI episodes, from -1 day (CRP, WBC) or -6 hours (vital signs) before to +8 days after the start of each episode, using box-cox transformed values as the outcome (normality assumption). Nonlinear trends were incorporated via natural cubic splines with four knots at the 20th, 40th, 60th and 80th percentiles of observed time values and included as fixed and random effects, as was the intercept (i.e. estimated vital sign/laboratory test value at the start of each episode). Fixed effects were included for baseline factors characterising each infection episode: source of infection, community-onset (blood sample collection ≤48 hours from admission), blood culture result (positive, potential contaminant, negative) and pathogen group (as in **Table S1**), age, sex, Charlson comorbidity and Elixhauser acuity scores, and immunosuppression. These covariates were selected based on clinical knowledge and prior literature, considering factors that may be associated with both the exposure (pathogen group or infection source) and the outcome (biomarker trajectories), and thus could potentially confound the observed associations. They were included regardless of statistical significance. Potential non-linear associations for age were considered via natural cubic splines with one knot at the 50th percentile (boundary knots at 5th and 95th) if it improved model fit (p<0.05). Interactions between each covariate and the natural cubic splines for time were assessed using likelihood ratio tests, comparing models with and without the interaction terms. Given the large number of interactions tested (36), to avoid multiple testing and collinearity problems, a p-value threshold of 0.05 was used to determine the inclusion of interaction terms in the final models, i.e. those with an impact on the trajectory as well as the baseline values (blood culture result and pathogen group, infection source, community-onset and immunosuppression). The final model had the form:

TimeSpline <- ns(df$time, knots = quantile(df$time, c(0.20, 0.40, 0.60, 0.80)), Boundary.knots = quantile(df$time, c(0.01, 0.99)))

RE_CRP <- lmer( CRP ~ Elixhauser + Charlson + Sex + ns(df$Age, knots = quantile(df$Age, c(0.50)), Boundary.knots = quantile(df$Age, c(0.05, 0.95)) + TimeSpline * (BugGroup + Source + CommunityOnset + ImmunoSuppression) + (TimeSpline | EpisodeID), data = df, REML = TRUE)

Separate adjusted models were fitted to examine effects of source of infection and baseline antimicrobial susceptibility. Models examining effects of source of infection were not adjusted for blood culture results because these would not be available for 24-48h but adjusted for other covariates above. Models examining baseline antimicrobial susceptibility were adjusted for blood culture results (i.e. pathogen group), source of infection and other covariates. These models included the main pathogen group covariate with categories as in **Table S1** and then an additional covariate which reflected “Susceptible”, “Resistant”, “No Antimicrobial Recorded” and “Unknown” for the Gram-positive, Gram-negative and other pathogens in **Table S1**. Effectively this was a partial interaction term, with no effect by definition for potential contaminants and culture-negative episodes, but for episodes with a pathogen, estimates that reflect the average effect of baseline antimicrobials being “Resistant”, “No Antimicrobial Recorded” or “Unknown” vs “Susceptible” across pathogens. This allowed us to fit a model that was nested within the main model, preserving the random effects structure.

Unadjusted latent class mixed models (LCMM) were used to identify underlying population-level heterogeneity in the response trajectories of routinely collected CRP measurements. We fitted LCMMs with between 1 and 6 classes, and the optimal number of classes was chosen based on the Bayesian Information Criterion (BIC) and percentage of class membership (all ≥0.5%) (**Table S5**).  This threshold was arbitrary, but was chosen based on our clinical judgment to balance model fit and the utility of the latent classes for understanding common CRP response patterns and guiding clinical decision-making. Patient characteristics and other covariates were not included in the models because we did not want to adjust for these potential causes of underlying heterogeneity but for the latent classes to reflect the underlying heterogeneity. From the selected LCMM, each episode was assigned to the class with the highest posterior probability. We then compared characteristics between the latent class groups univariably. The Standardised Mean Difference (SMD) was used in the comparison given the large sample size, and to better account for differences in measurement scales. We did not employ the three-step approach^4^ to test class membership predictors as available package (lcmm 2.0.0) did not support this methodology^5^.

Centile reference charts for expected CRP response in standard responders (i.e. those with peak response on day-1/2) were constructed using the lambda-mu-sigma (LMS) method^6^, adopted by the World Health Organization (WHO) to generate the childhood growth standards^7,8^, and implemented using Generalised Additive Models for Location Scale and Shape library (GAMLSS, version 5.4.3)^9^. Three candidate distributions were considered: Box-Cox Cole Green (truncated standard normal distribution), Box-Cox power exponential (truncated exponential power distribution), and Box-Cox t (truncated t distribution), selected using the Generalised Akaike Information Criterion^10^, with degrees of freedom for the penalised spline model smoothing parameters determined using the local Schwarz Bayesian Criterion^10^. With these parameters, z-scores and percentiles of CRP response can be generated for times after blood culture collection.

The LMS method assumes the measurements used for constructing centile charts are independent, whereas serial measurements of vital signs and laboratory tests are correlated within individuals^11^. This could potentially cause bias, especially if patients with abnormal responses get prolonged and more frequent measurements. The common solution of selecting one random observation for each patient can result in significant information loss. We addressed the problem of potentially informative numbers of measurements by first conducting sensitivity analyses selecting one random observation per episode, and second by estimating centiles by constructing 100,000 bootstrap samples of the 40,620 episodes from standard responders and using a linear mixed model with only fixed and random effects for time (natural cubic spline) as above to simulate the CRP values of these patients at nine random time points up to 8 days after the start of each episode (incorporating fixed effects, correlated random effects per patient and additional measurement error in the prediction) then using these values (100,000 episodes, 900,000 observations) as outcomes for the LMS model.

Analyses were performed using statistical software R, version 4.1.0 (R Project for Statistical Computing). Model fitting was performed using the ‘lme4’ package (version 1.1-27.1) (using restricted maximum likelihood estimation (REML) with the ‘t-tests use Satterthwaite’s method’ approach for approximating degrees of freedom, as implemented in the ‘lmerModLmerTest’ function), the ‘lcmm’ package (version 2.1.0), and the ‘gamlss’ package (version 5.4-20). The R code for our analysis is available at github.com/guqingze/bsi_normal_response.

# Supplementary Results

## Model Diagnostics

We considered residual checks using DHARMa for the main linear mixed model of box-cox transformed CRP response trajectories, and calculation of marginal and conditional R-squared values using MuMIn. The QQ plots showed that the residuals were approximately normally distributed (**Figure S13**). The marginal R-squared value of 0.160 indicated that the fixed effects in our model accounted for 16% of the variability in CRP trajectories. The conditional R-squared value of 0.971 suggested that the combination of fixed and random effects explains a substantial proportion of the total variability in CRP trajectories, expected since we included random effects on both the intercept (baseline CRP) and the non-linear time trajectories for each BSI episode, allowing individual heterogeneity to be captured.

## Distribution of centile changes between two consecutive days

To assess the extent to which individual patients' CRP trajectories follow the population-level centile charts within a BSI episode, we calculated the distribution of centile changes between consecutive days for episodes with available CRP measurements (**Figure 4**). Focusing on the recovery stage from day 2 onwards, we found that 90–96% of episodes had centile changes ≤25 between consecutive days, 84-93% ≤20 and 65-79% ≤10. This finding suggests that most patients' response trajectories track along the centile curves, supporting the use of these charts to monitor individual patient progress and to identify deviations from the expected recovery pattern.

# Supplementary Tables

| **Blood Culture Results** | **CRP Measured at least  once during the episode**, N = 77,957 (88.2%)^1^ | **CRP Not Measured**, N = 10,391 (11.8%)^1^ | **Standardised Difference**^2^ |
| --- | --- | --- | --- |
| Gram-positive Pathogens | | | |
| *Staphylococcus aureus* | 697 (0.9%) | 19 (0.2%) | 0.10 |
| Beta-Hemolytic Streptococci | 388 (0.5%) | 31 (0.3%) | 0.03 |
| *Enterococcus* sp. | 329 (0.4%) | 14 (0.1%) | 0.05 |
| *Streptococcus pneumoniae* | 290 (0.4%) | 16 (0.2%) | 0.04 |
| Other Pathogenic *Streptococcus* | 124 (0.2%) | 4 (0.0%) | 0.04 |
| Gram-negative Pathogens | | | |
| *Escherichia coli* | 2,284 (2.9%) | 126 (1.2%) | 0.12 |
| *Klebsiella* sp. | 503 (0.6%) | 21 (0.2%) | 0.07 |
| Other *Enterobacterales* | 349 (0.4%) | 18 (0.2%) | 0.05 |
| *Pseudomonas aeruginosa* | 289 (0.4%) | 14 (0.1%) | 0.05 |
| *Enterobacter* sp. | 127 (0.2%) | 5 (0.0%) | 0.04 |
| Other Pathogens | | | |
| Other | 510 (0.7%) | 45 (0.4%) | 0.03 |
| Polymicrobial | 309 (0.4%) | 6 (0.1%) | 0.07 |
| Anaerobes | 284 (0.4%) | 23 (0.2%) | 0.03 |
| *Candida* sp. | 81 (0.1%) | 2 (0.0%) | 0.03 |
| Potential Contaminant(s) and Culture-negative | | | |
| Culture-negative | 67,464 (86.5%) | 9,667 (93.0%) | 0.22 |
| CoNS (contaminant) | 3,184 (4.1%) | 305 (2.9%) | 0.06 |
| Other Suspected Contaminants | 441 (0.6%) | 59 (0.6%) | 0.00 |
| Viridans and Other *Streptococcus* | 304 (0.4%) | 16 (0.1%) | 0.05 |
| ^1^n (%) | | | |
| ^2^Standardised Mean Difference | | | |

**Table S1.** Comparison of blood culture results for suspected BSIs episodes with versus without ≥1 measurement of CRP within 1 day before to 8 days after the start of each episode. Other Pathogenic *Streptococcus* includes *Streptococcus anginosus*, *Streptococcus gallolyticus*, *Streptococcus constellatus*, *Streptococcus intermedius*, *Streptococcus lutetiensis*, *Streptococcus bovis*. CoNS (contaminant) refers to Coagulase negative staphylococci. Percentages in the header are of all episodes, and in the main body are column percentages within each group. Effect size estimated using standardised mean difference (SMD), considering 0.2, 0.5, and 0.8 as small, medium, and large, respectively.

|  | Baseline Antimicrobial Susceptibility | | | | |
| --- | --- | --- | --- | --- | --- |
|  | Susceptible | Resistant | No Antimicrobial Recorded | Unknown | Total |
| Gram-positive Pathogens | | | | | |
| *Staphylococcus aureus* | 601 (86%) | 26 (3.7%) | 67 (9.6%) | 3 (0.4%) | 697 (100%) |
| Beta-Hemolytic Streptococci | 372 (96%) | 0 (0%) | 14 (3.6%) | 2 (0.5%) | 388 (100%) |
| *Enterococcus* sp. | 224 (68%) | 53 (16%) | 49 (15%) | 3 (0.9%) | 329 (100%) |
| *Streptococcus pneumoniae* | 278 (96%) | 0 (0%) | 11 (3.8%) | 1 (0.3%) | 290 (100%) |
| Other Pathogenic *Streptococcus* | 101 (81%) | 2 (1.6%) | 20 (16%) | 1 (0.8%) | 124 (100%) |
| Gram-negative Pathogens | | | | | |
| *Escherichia coli* | 1,874 (82%) | 342 (15%) | 65 (2.8%) | 3 (0.1%) | 2,284 (100%) |
| *Klebsiella* sp. | 411 (82%) | 54 (11%) | 35 (7.0%) | 3 (0.6%) | 503 (100%) |
| Other *Enterobacterales* | 280 (80%) | 43 (12%) | 23 (6.6%) | 3 (0.9%) | 349 (100%) |
| *Pseudomonas aeruginosa* | 177 (61%) | 82 (28%) | 27 (9.3%) | 3 (1.0%) | 289 (100%) |
| *Enterobacter* sp. | 88 (69%) | 30 (24%) | 9 (7.1%) | 0 (0%) | 127 (100%) |
| Other Pathogens | | | | | |
| Other | 215 (42%) | 64 (13%) | 74 (15%) | 157 (31%) | 510 (100%) |
| Polymicrobial | 170 (55%) | 92 (30%) | 32 (10%) | 15 (4.9%) | 309 (100%) |
| Anaerobes | 222 (78%) | 32 (11%) | 27 (9.5%) | 3 (1.1%) | 284 (100%) |
| *Candida* sp. | 4 (4.9%) | 64 (79%) | 12 (15%) | 1 (1.2%) | 81 (100%) |
| Total | 5,017 (76%) | 884 (13%) | 465 (7.1%) | 198 (3.0%) | 6,564 (100%) |

**Table S2.** Distribution of baseline antimicrobial susceptibility across pathogen groups for suspected BSI episodes with ≥1 measurement of CRP within 1 day before to 8 days after the start of each episode. Culture-negative episodes and episodes with potential contaminants were excluded. See supplemental methods for definition of baseline antimicrobial susceptibility. Note: Baseline antimicrobials include both antibiotics and antifungals.

|  |  | | **Comparisons with Peak on Day 1** | | | | | | | |
| --- | --- | --- | --- | --- | --- | --- | --- | --- | --- | --- |
| **Characteristic** | | **Peak on Day 1** | **Peak on Day 2** | **Difference**^2^ | **Slow Recovery** | **Difference**^2^ | **Peak on Day 6** | **Difference**^2^ | **Low Response** | **Difference**^2^ |
| Age at admission (years) | | 69.2 (51.6, 81.1) | 68.4 (46.7, 81.7) | 0.06 | 70.3 (56.4, 81.0) | 0.13 | 70.1 (56.0, 81.6) | 0.11 | 63.6 (43.6, 79.3) | 0.19 |
| Sex (Male) | | 18,865 (52.3%) | 2,240 (49.5%) | 0.06 | 6,178 (57.9%) | 0.11 | 415 (55.9%) | 0.07 | 11,363 (43.8%) | 0.17 |
| Charlson score | | 1 (0, 2) | 1 (0, 2) | 0.01 | 1 (1, 3) | 0.16 | 2 (1, 3) | 0.20 | 1 (0, 2) | 0.04 |
| Elixhauser score | | 2 (1, 4) | 2 (1, 4) | 0.01 | 3 (2, 4) | 0.20 | 3 (2, 4) | 0.22 | 2 (1, 4) | 0.04 |
| Community-onset | | 28,184 (78.1%) | 4,010 (88.5%) | 0.28 | 7,162 (67.1%) | 0.25 | 521 (70.1%) | 0.18 | 22,189 (85.6%) | 0.20 |
| Immunosuppression | | 4,909 (13.6%) | 533 (11.8%) | 0.06 | 2,222 (20.8%) | 0.19 | 164 (22.1%) | 0.22 | 3,797 (14.6%) | 0.03 |
| Diabetes mellitus | | 7,225 (20.0%) | 921 (20.3%) | 0.01 | 2,335 (21.9%) | 0.05 | 158 (21.3%) | 0.03 | 4,862 (18.8%) | 0.03 |
| Palliative care | | 1,995 (5.5%) | 200 (4.4%) | 0.05 | 1,332 (12.5%) | 0.24 | 79 (10.6%) | 0.19 | 892 (3.4%) | 0.10 |
| >1 blood cultures in episode | | 9,386 (26.0%) | 1,792 (39.6%) | 0.29 | 6,096 (57.2%) | 0.67 | 495 (66.6%) | 0.89 | 4,688 (18.1%) | 0.19 |
| >1 positive blood cultures in episode | | 706 (2.0%) | 136 (3.0%) | 0.07 | 663 (6.2%) | 0.22 | 27 (3.6%) | 0.10 | 191 (0.7%) | 0.11 |
| Baseline antimicrobial susceptibility | |  |  | 0.20 |  | 0.20 |  | 0.30 |  | 0.32 |
| Culture-negative | | 30,916 (85.7%) | 3,550 (78.4%) |  | 8,352 (78.3%) |  | 642 (86.4%) |  | 24,004 (92.6%) |  |
| Potential contaminant(s) | | 1,672 (4.6%) | 245 (5.4%) |  | 673 (6.3%) |  | 65 (8.7%) |  | 1,274 (4.9%) |  |
| Susceptible | | 2,795 (7.7%) | 606 (13.4%) |  | 1,220 (11.4%) |  | 17 (2.3%) |  | 379 (1.5%) |  |
| Resistant | | 447 (1.2%) | 82 (1.8%) |  | 235 (2.2%) |  | 8 (1.1%) |  | 112 (0.4%) |  |
| No antimicrobial recorded | | 176 (0.5%) | 28 (0.6%) |  | 147 (1.4%) |  | 6 (0.8%) |  | 108 (0.4%) |  |
| Unknown | | 85 (0.2%) | 18 (0.4%) |  | 39 (0.4%) |  | 5 (0.7%) |  | 51 (0.2%) |  |
| ^1^Median (IQR); n (%); ^2^Standardised Mean Difference | | | | | | | | | | |

**Table S3.** Comparison of episode characteristics between those estimated from latent class models as Peak on Day 2, Slow Recovery, Peak on Day 6, Low Response, and Peak on Day 1 as the reference group. Unadjusted effect size estimated using standardised mean difference (SMD), considering 0.2, 0.5, and 0.8 as small, medium, and large, respectively.

| **Characteristic** | **OR**^1^ | **95% CI**^1^ | **p-value** |
| --- | --- | --- | --- |
| Age at admission (10 years) | 1.24 | 1.21, 1.27 | **<0.001** |
| Sex (male vs female) | 1.02 | 0.96, 1.09 | 0.52 |
| Charlson score | 0.97 | 0.94, 1.00 | 0.10 |
| Elixhauser score | 1.04 | 1.02, 1.06 | **<0.001** |
| NEWS score (baseline) | 1.19 | 1.17, 1.20 | **<0.001** |
| Immunosuppression | 0.91 | 0.84, 0.99 | **0.028** |
| Palliative care | 5.85 | 5.40, 6.35 | **<0.001** |
| Community-onset | 0.80 | 0.75, 0.87 | **<0.001** |
| Latent CRP trajectory class |  |  |  |
| Peak on Day 1 | — | — |  |
| Peak on Day 2 | 0.89 | 0.78, 1.03 | 0.12 |
| Slow Recovery | 2.00 | 1.85, 2.17 | **<0.001** |
| Peak on Day 6 | 1.95 | 1.50, 2.51 | **<0.001** |
| Low Response | 0.76 | 0.71, 0.83 | **<0.001** |
| Blood culture result |  |  |  |
| E. coli | — | — |  |
| Klebsiella sp. | 1.09 | 0.76, 1.55 | 0.64 |
| Other Enterobacterales | 1.22 | 0.81, 1.82 | 0.34 |
| Pseudomonas aeruginosa | 1.35 | 0.88, 2.05 | 0.17 |
| Enterobacter sp. | 1.29 | 0.63, 2.58 | 0.48 |
| Staphylococcus aureus | 1.66 | 1.20, 2.29 | **0.002** |
| Beta-Hemolytic Streptococci | 1.02 | 0.65, 1.58 | 0.93 |
| Enterococcus sp. | 1.29 | 0.86, 1.94 | 0.22 |
| Streptococcus pneumoniae | 0.97 | 0.58, 1.60 | 0.92 |
| Other Pathogenic Streptococcus | 0.78 | 0.37, 1.59 | 0.51 |
| Other | 1.62 | 1.12, 2.35 | **0.010** |
| Polymicrobial | 1.14 | 0.77, 1.67 | 0.52 |
| Anaerobes | 1.85 | 1.13, 3.02 | **0.014** |
| Candida sp. | 2.05 | 0.98, 4.19 | 0.053 |
| Culture-negative | 1.05 | 0.89, 1.23 | 0.60 |
| CoNS (contaminant) | 1.25 | 1.01, 1.54 | **0.040** |
| Other Suspected Contaminants | 0.97 | 0.60, 1.53 | 0.89 |
| Viridans and Other Streptococcus | 0.99 | 0.59, 1.62 | 0.96 |
| Source of infection |  |  |  |
| Urinary | — | — |  |
| Unspecific | 1.62 | 1.43, 1.84 | **<0.001** |
| Respiratory | 1.72 | 1.53, 1.93 | **<0.001** |
| Multiple sources | 1.42 | 1.25, 1.62 | **<0.001** |
| Abdominal | 1.58 | 1.35, 1.85 | **<0.001** |
| Skin, soft tissue, orthopedic | 0.91 | 0.75, 1.10 | 0.32 |
| CNS | 3.53 | 2.40, 5.13 | **<0.001** |
| Other | 1.19 | 0.87, 1.61 | 0.27 |
| ^1^OR = Odds Ratio, CI = Confidence Interval | | | |

**Table S4.** Independent predictors of 30-day all-cause mortality. Baseline NEWS score was calculated using the closest set of vital signs within 1 day before to 1 day after the start of each episode.

| **N classes** | **Log likelihood** | **AIC** | **BIC** | **SABIC** | **Entropy** | **Class 1 (%)** | **Class 2 (%)** | **Class 3 (%)** | **Class 4 (%)** | **Class 5 (%)** | **Class 6 (%)** |
| --- | --- | --- | --- | --- | --- | --- | --- | --- | --- | --- | --- |
| 1 | -522381.4 | 1044804.9 | 1044999.4 | 1044932.7 | 1.000 | 100.0 |  |  |  |  |  |
| 2 | -505915.1 | 1011884.3 | 1012134.4 | 1012048.6 | 0.856 | 0.7 | 99.3 |  |  |  |  |
| 3 | -499823.5 | 999713.0 | 1000018.7 | 999913.8 | 0.598 | 5.5 | 61.2 | 33.2 |  |  |  |
| 4 | -499309.0 | 998695.9 | 999057.2 | 998933.3 | 0.645 | 0.5 | 32.6 | 61.3 | 5.6 |  |  |
| 5 | -499012.2 | 998114.3 | 998531.2 | 998388.2 | 0.505 | 1.0 | 13.7 | 46.3 | 33.3 | 5.8 |  |
| 6 | -498126.1 | 996354.2 | 996826.7 | 996664.6 | 0.542 | 0.4 | 7.1 | 37.7 | 25.9 | 25.9 | 3.0 |

**Table S5.** Fit indices for latent class mixed models with different numbers of classes. Abbreviations: AIC: Akaike Information Criterion; BIC: Bayesian Information Criterion; SABIC: Sample-size adjusted BIC.

# Supplementary Figures


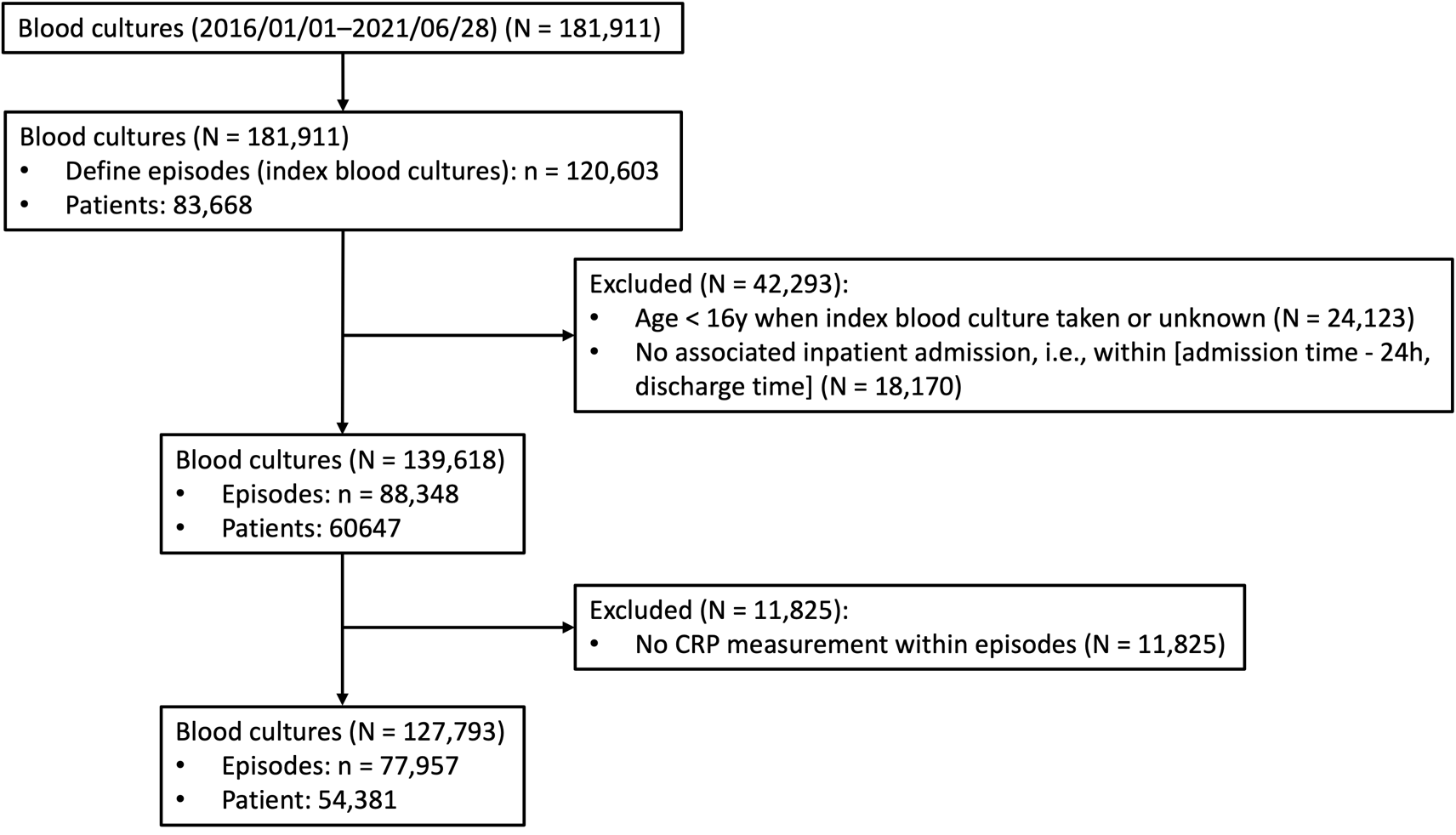


**Figure S1.** Flowchart for identifying suspected BSI infections and selecting the dataset for outcome investigations. *Index blood culture: the first blood sample from which a microbial pathogen was cultured, otherwise the first blood sample from which a contaminant was identified, or otherwise the first blood sample (if all cultures were negative). Note: For those excluded due to timing of index blood cultures, 70.0%[4,468/6,403] were culture-negative samples taken >24h before admission.


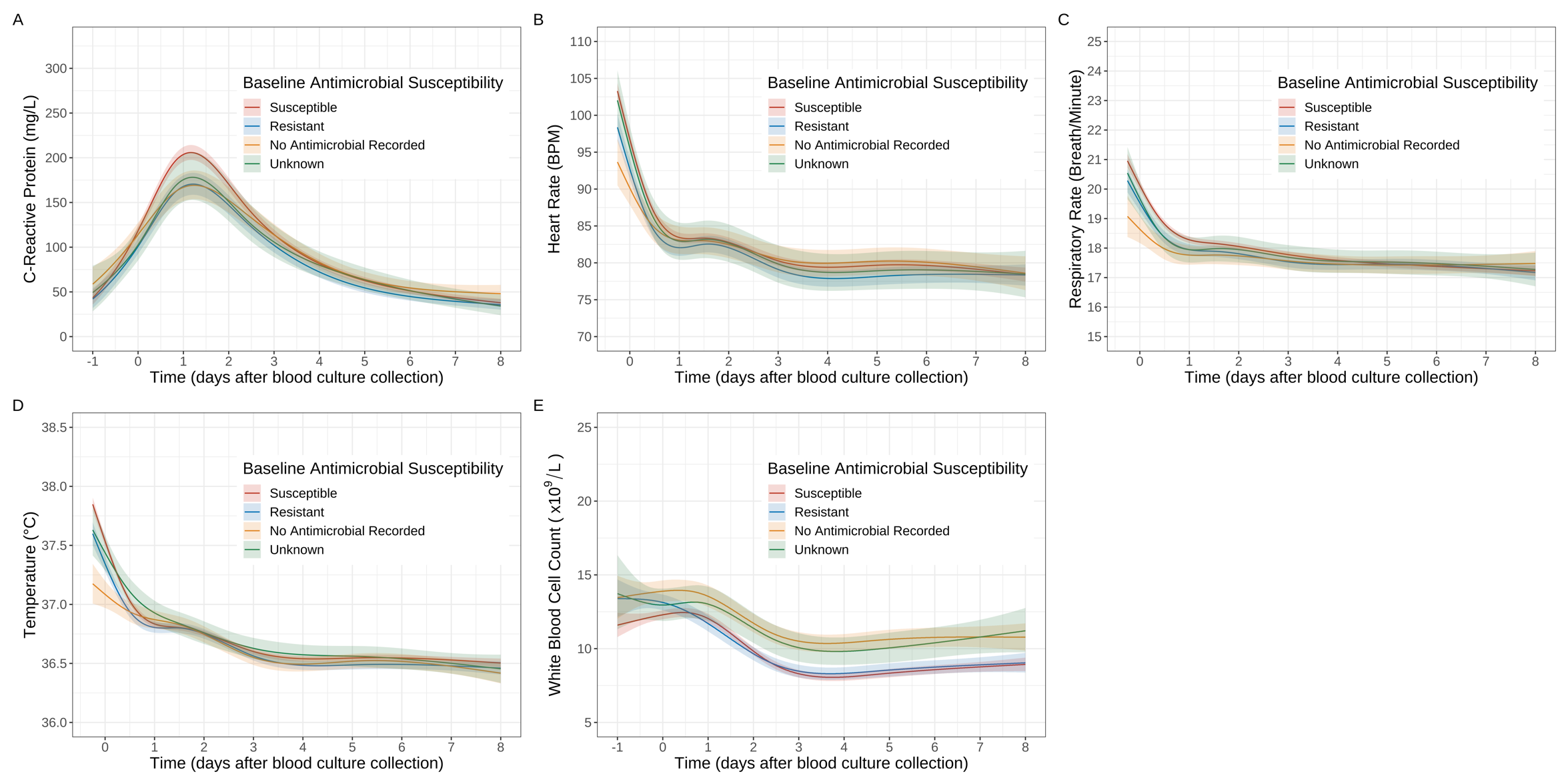


**Figure S2.** Response trajectories of CRP (A), heart rate (B), respiratory rate (C), body temperature (D) and WBC count (E) following different baseline antimicrobial susceptibilities. Predictions are plotted at the reference values of other adjusting variables: age = 64 years, male, Charlson score = 1, Elixhauser score = 3, community-onset, absence of immunosuppression, urinary source, and *E. coli* infection. Note: the no antimicrobial recorded group were enriched for people who were sufficiently well that they might not have received antimicrobials at baseline.


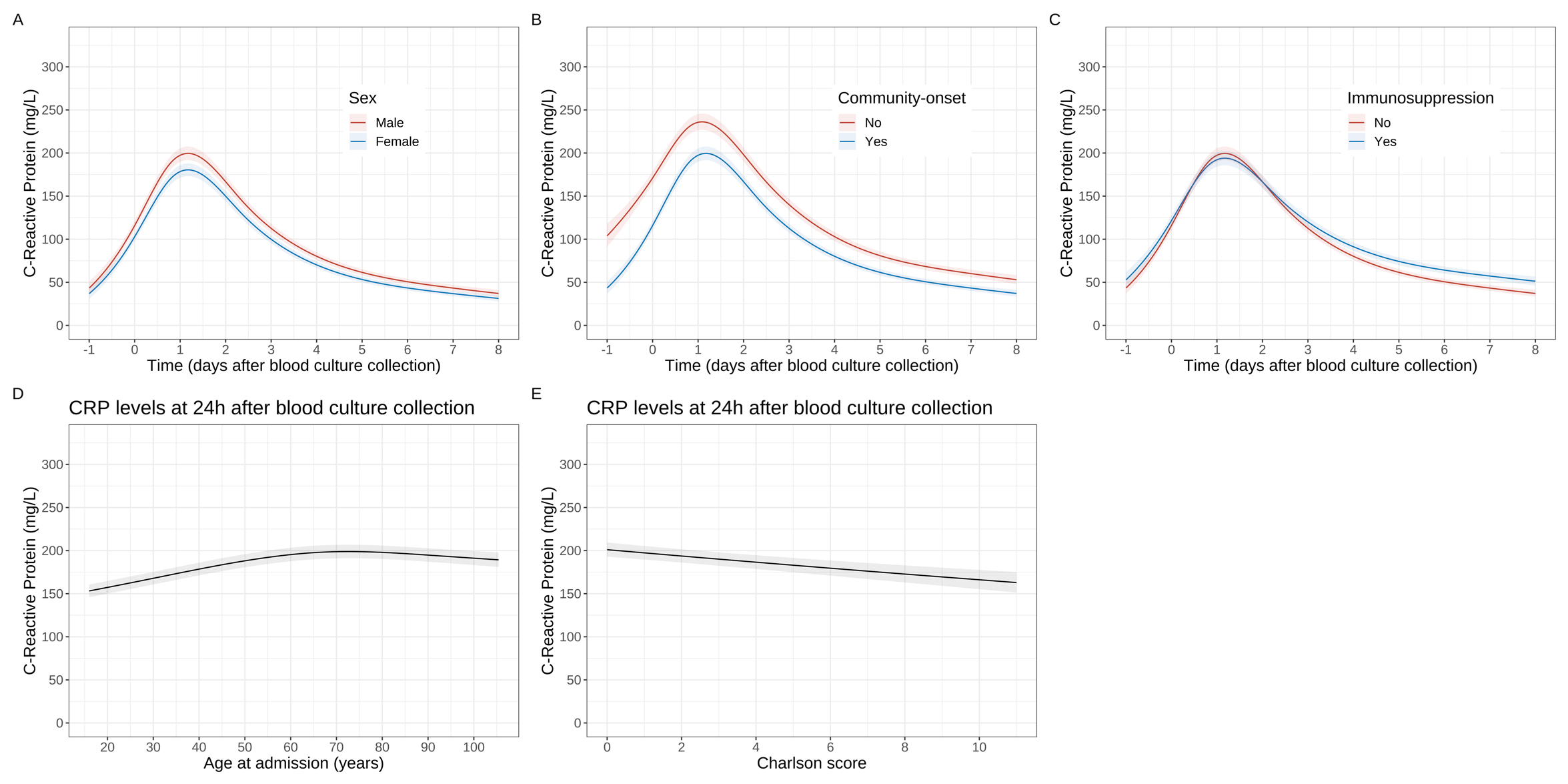


**Figure S3.** The adjusted associations between CRP response and sex (A), community-onset (B), immunosuppression (C), age (D) and Charlson score (E). Panels A–C present the relationships between CRP response trajectories from 1 days before to 8 days after the start of each episode. Panels D–E display CRP levels at 24 hours after the start of each episode to demonstrate the association between these continuous covariates and peak CRP levels (no evidence of interaction between these covariates and time; no evidence of association between Elixhauser and peak levels). Predictions are plotted at the reference values of other variables: age = 64 years, male, Charlson score = 1, Elixhauser score = 3, community-onset, absence of immunosuppression, urinary source, and *E. coli* infection. Nonlinear trends were incorporated via natural cubic splines with four knots at the 20th, 40th, 60th and 80th percentiles of observed time values (day 0, day 0.8, day 2.4, day 4.7).


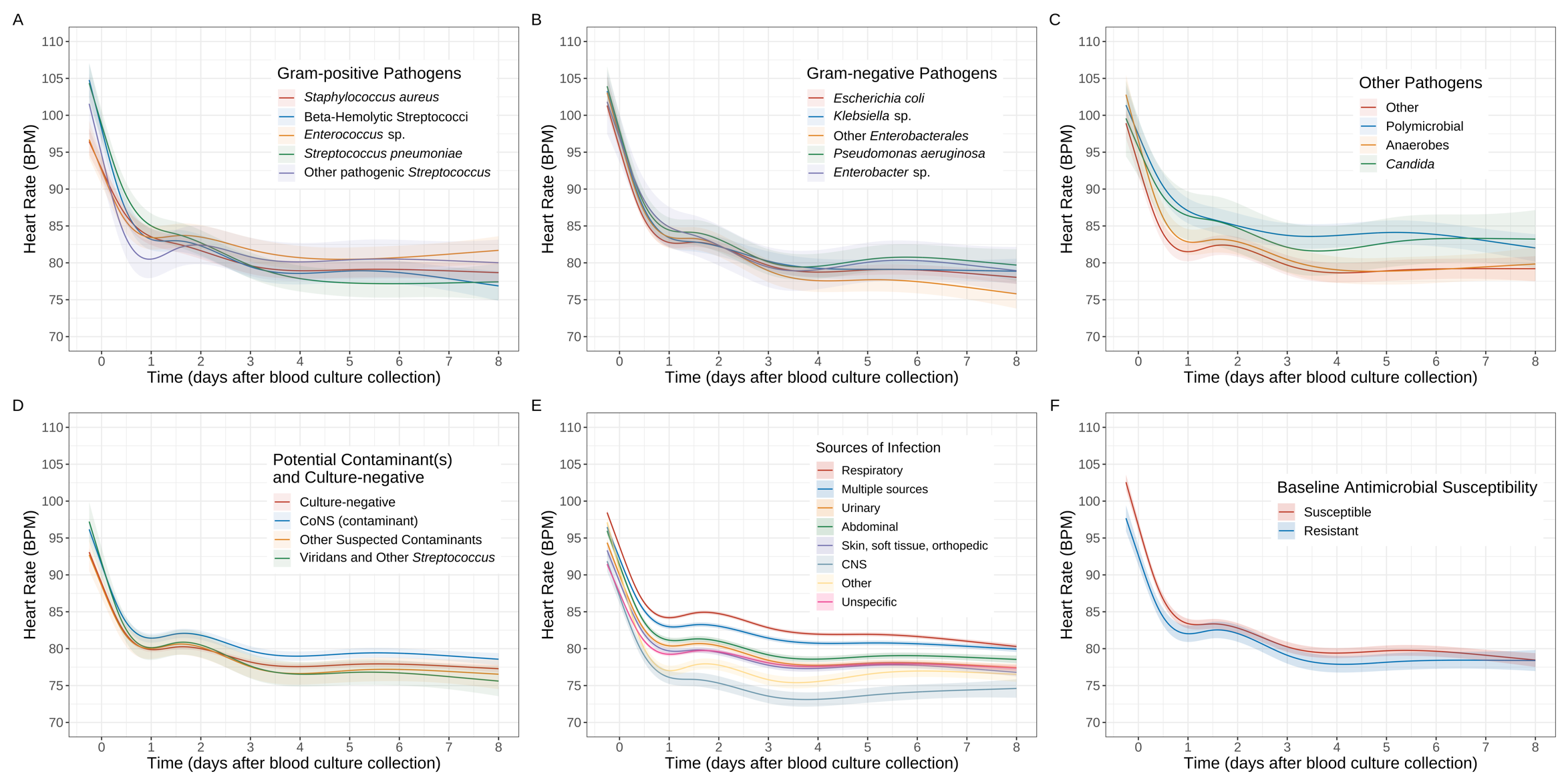


**Figure S4.** Heart rate response trajectories following different blood culture results (Gram-positive pathogens (A), Gram-negative pathogens (B), other pathogens (C), and potential contaminants and culture-negative results (D); adjusted for source of infection and other covariates), sources of infection (E) (not adjusted for blood culture results but adjusted for other covariates) and baseline antimicrobial susceptibilities (F) (adjusted for blood culture results, source of infection and other covariates). See **Figure S2B** for response trajectories of no baseline antimicrobial recorded and unknown baseline susceptibility. Predictions are plotted at the reference values of other adjusting variables: age = 64 years, male, Charlson score = 1, Elixhauser score = 3, community-onset, absence of immunosuppression, urinary source (excluding panel E), and *E. coli* infection (panel F only). Modelling time was limited to 6 hours prior to the start of each episode, as vital signs were not frequently measured before this point. Nonlinear trends were incorporated via natural cubic splines with four knots at the 20th, 40th, 60th and 80th percentiles of observed time values (day 0.4, day 1.5, day 3.0, day 5.1).


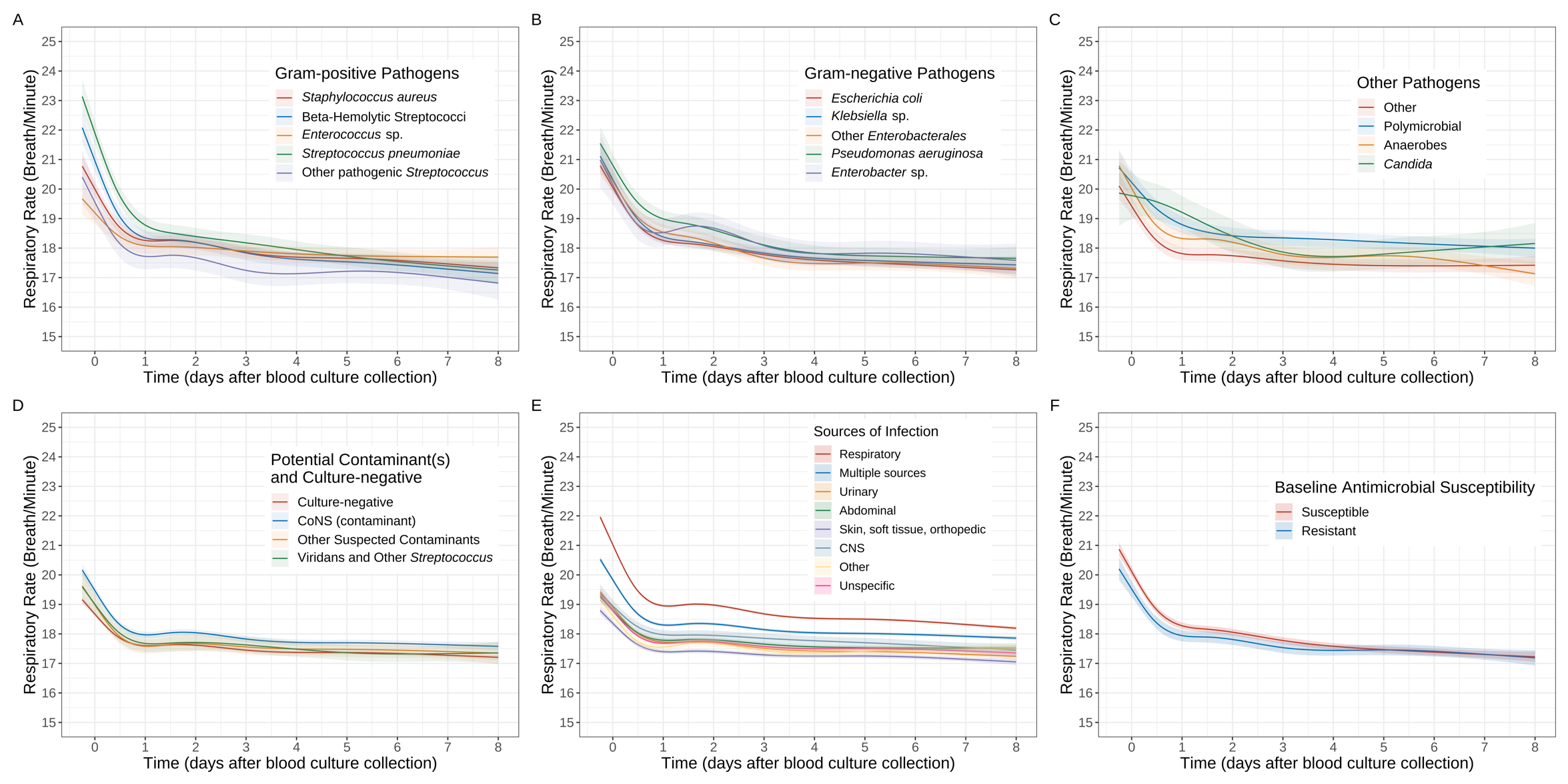


**Figure S5.** Respiratory rate response trajectories following different blood culture results (Gram-positive pathogens (A), Gram-negative pathogens (B), other pathogens (C), and potential contaminants and culture-negative results (D); adjusted for source of infection and other covariates), sources of infection (E) (not adjusted for blood culture results but adjusted for other covariates) and baseline antimicrobial susceptibilities (F) (adjusted for blood culture results, source of infection and other covariates). See **Figure S2C** for response trajectories of no baseline antimicrobial recorded and unknown baseline susceptibility. Predictions are plotted at the reference values of other adjusting variables: age = 64 years, male, Charlson score = 1, Elixhauser score = 3, community-onset, absence of immunosuppression, urinary source (excluding panel E), and *E. coli* infection (panel F only). Modelling time was limited to 6 hours prior to the start of each episode, as vital signs were not frequently measured before this point. Nonlinear trends were incorporated via natural cubic splines with four knots at the 20th, 40th, 60th and 80th percentiles of observed time values (day 0.4, day 1.5, day 3.0, day 5.1).


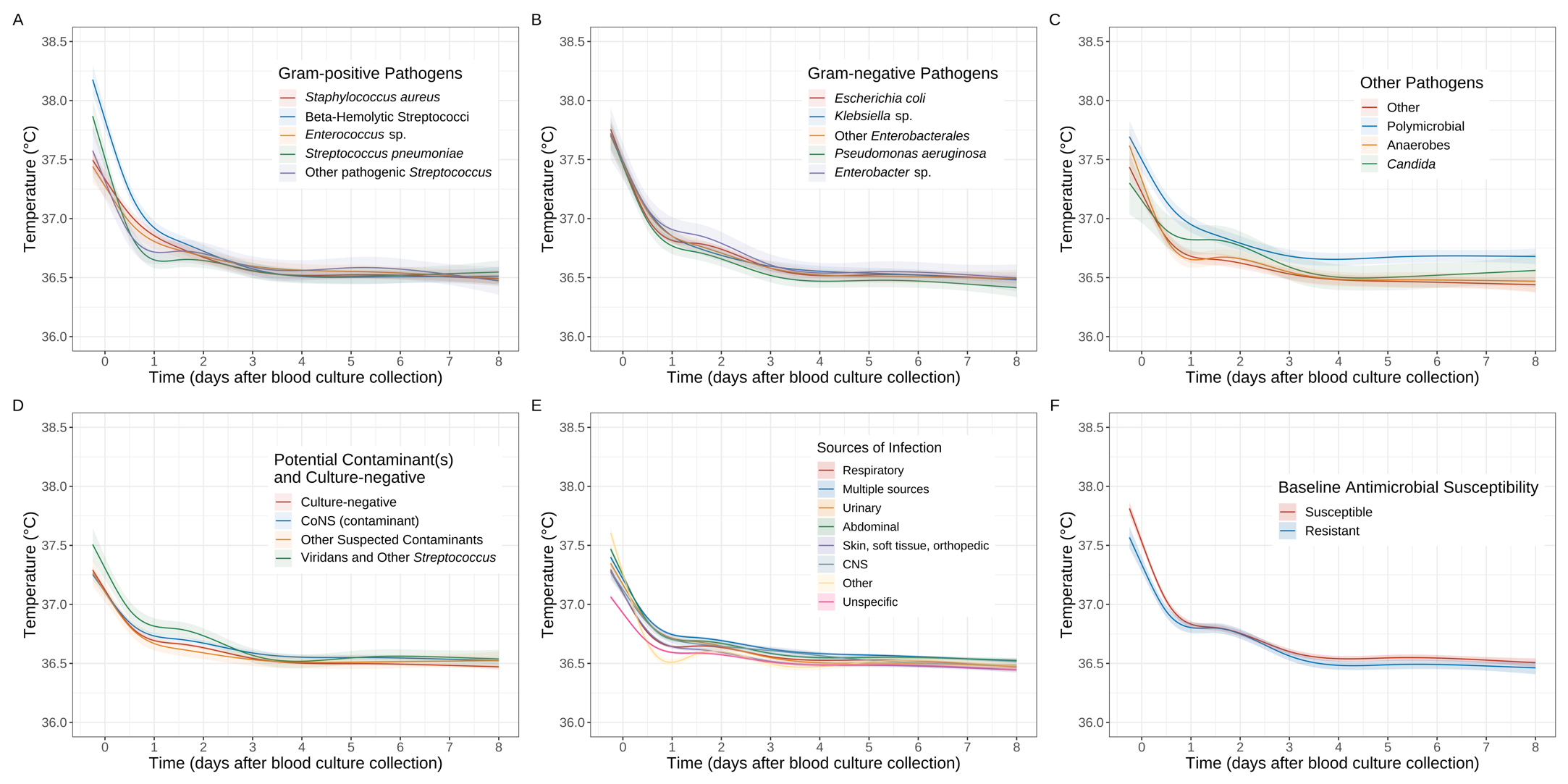


**Figure S6.** Body temperature response trajectories following different blood culture results (Gram-positive pathogens (A), Gram-negative pathogens (B), other pathogens (C), and potential contaminants and culture-negative results (D); adjusted for source of infection and other covariates), sources of infection (E) (not adjusted for blood culture results but adjusted for other covariates) and baseline antimicrobial susceptibilities (F) (adjusted for blood culture results, source of infection and other covariates). See **Figure S2D** for response trajectories of no baseline antimicrobial recorded and unknown baseline susceptibility. The acute temperature response with “other” source of infection in panel E (bright yellow) was potentially driven by brisk immune responses in younger patients with ENT and obstetric infections (and/or potential overfitting in this relatively small group, **Table 1**). Predictions are plotted at the reference values of other adjusting variables: age = 64 years, male, Charlson score = 1, Elixhauser score = 3, community-onset, absence of immunosuppression, urinary source (excluding panel E), and *E. coli* infection (panel F only). Modelling time was limited to 6 hours prior to the start of each episode, as vital signs were not frequently measured before this point. Nonlinear trends were incorporated via natural cubic splines with four knots at the 20th, 40th, 60th and 80th percentiles of observed time values (day 0.5, day 1.5, day 3.0, day 5.1).


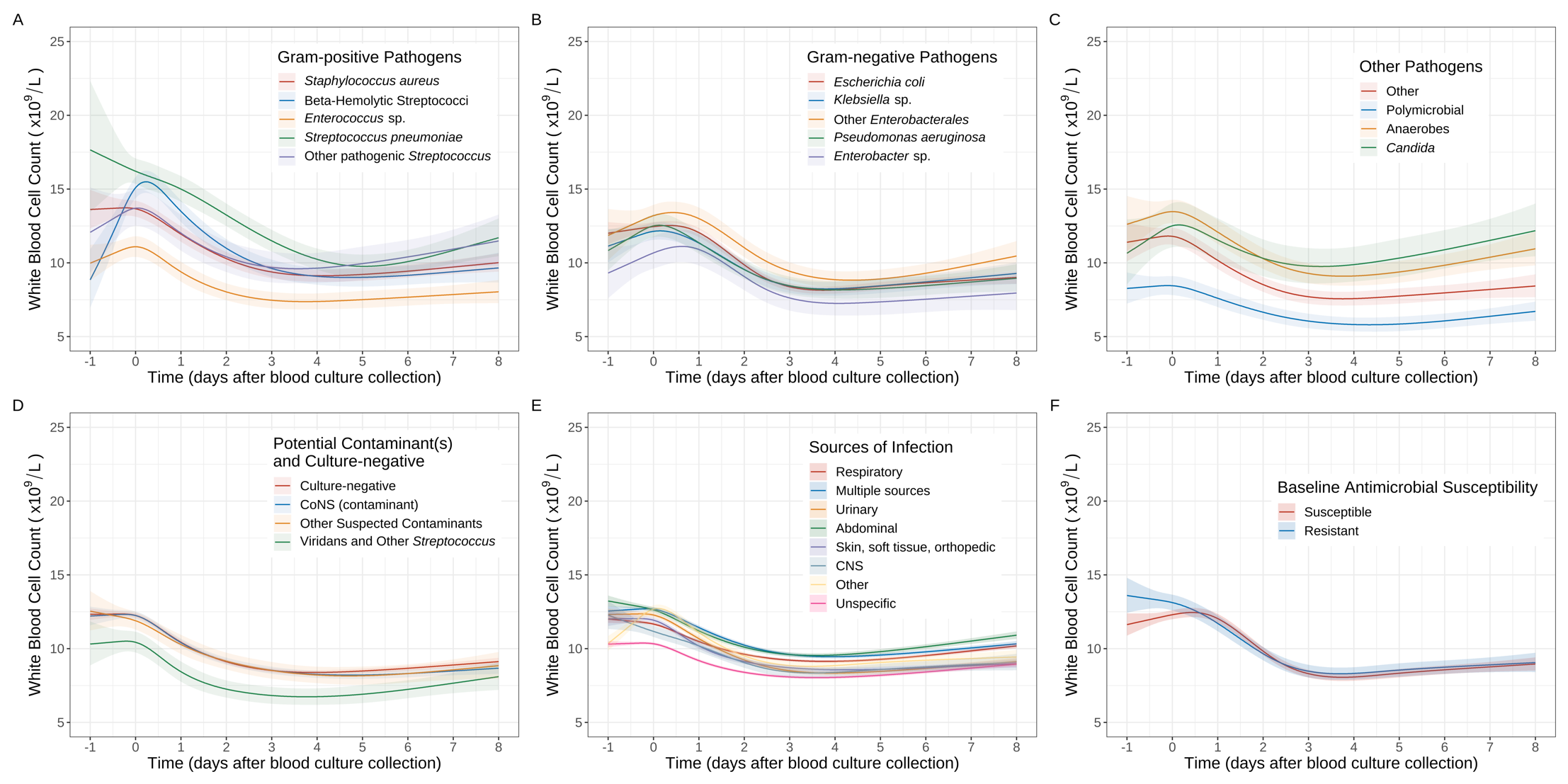


**Figure S7.** WBC count response trajectories following different blood culture results (Gram-positive pathogens (A), Gram-negative pathogens (B), other pathogens (C), and potential contaminants and culture-negative results (D); adjusted for source of infection and other covariates), sources of infection (E) (not adjusted for blood culture results but adjusted for other covariates) and baseline antimicrobial susceptibilities (F) (adjusted for blood culture results, source of infection and other covariates). See **Figure S2E** for response trajectories of no baseline antimicrobial recorded and unknown baseline susceptibility. The acute WBC count response with “other” source of infection in panel E (bright yellow) was potentially driven by brisk immune responses in younger patients with ENT and obstetric infections (and/or potential overfitting in this relatively small group, **Table 1**). Predictions are plotted at the reference values of other adjusting variables: age = 64 years, male, Charlson score = 1, Elixhauser score = 3, community-onset, absence of immunosuppression, urinary source (excluding panel E), and *E. coli* infection (panel F only). Nonlinear trends were incorporated via natural cubic splines with four knots at the 20th, 40th, 60th and 80th percentiles of observed time values (day 0, day 0.8, day 2.4, day 4.7).


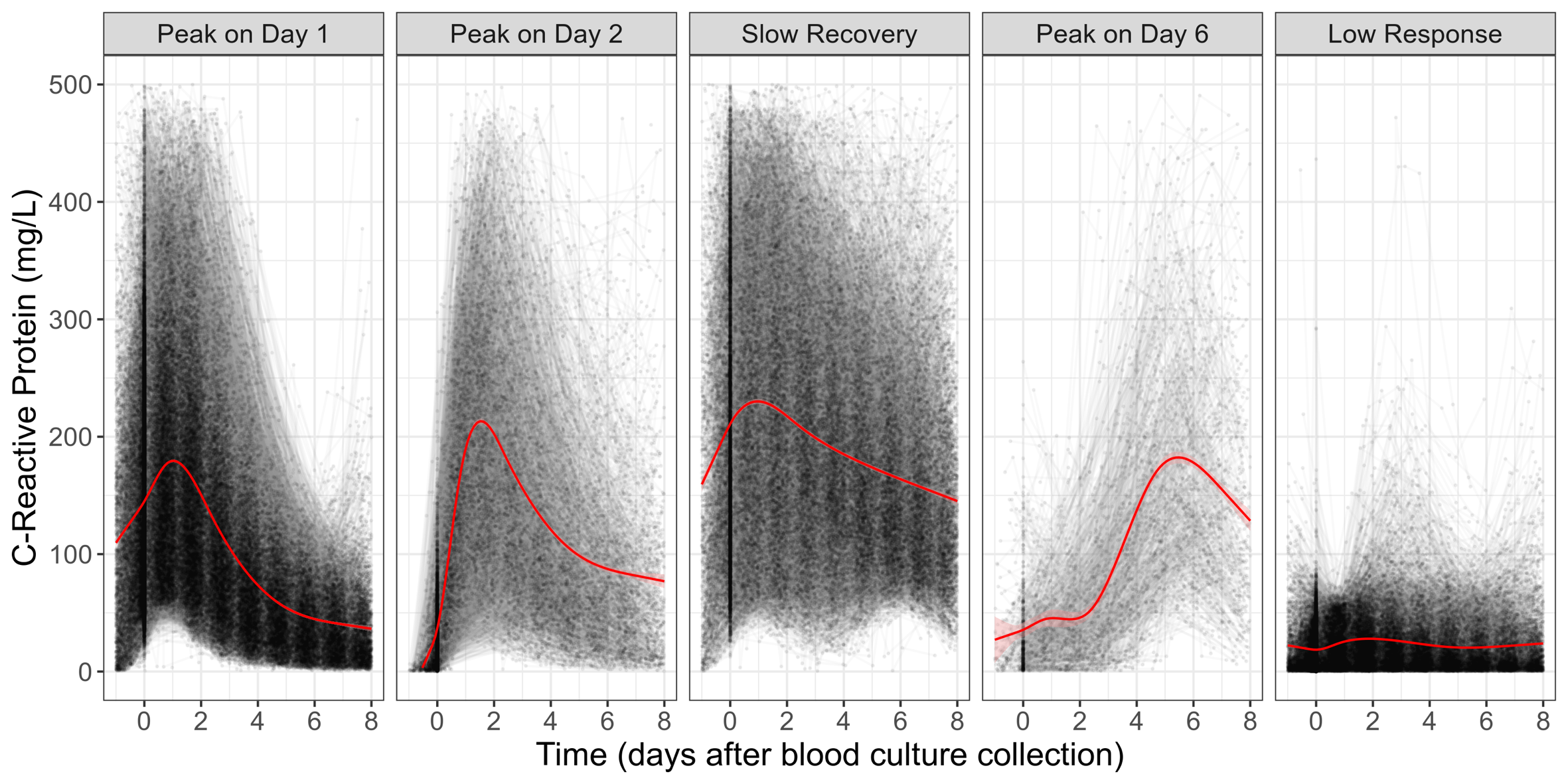


**Figure S8.** Spaghetti plot illustrating raw data underpinning latent classes of CRP response trajectories. Black lines represent individual CRP response trajectories for all suspected BSIs episodes with CRP measurements, while red lines show the mean response trajectory for each latent trajectory class.


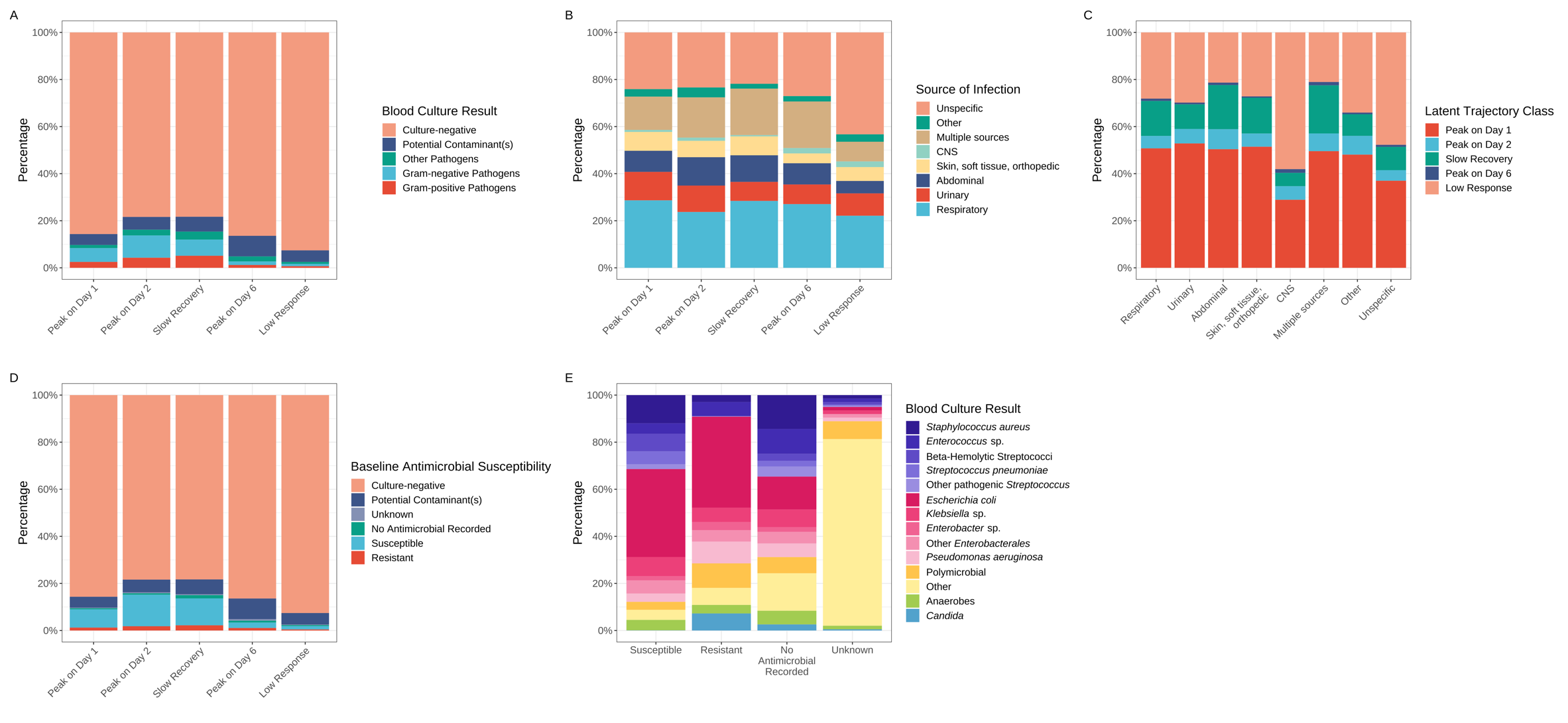


**Figure S9.** Associations between latent trajectory classes of the CRP response and blood culture results (A), sources of infection (B, C), baseline antimicrobial susceptibility (D), and pathogen groups' association with baseline antimicrobial susceptibility (E). See **Table S2** for cross table of pathogen groups versus baseline antimicrobial susceptibility.


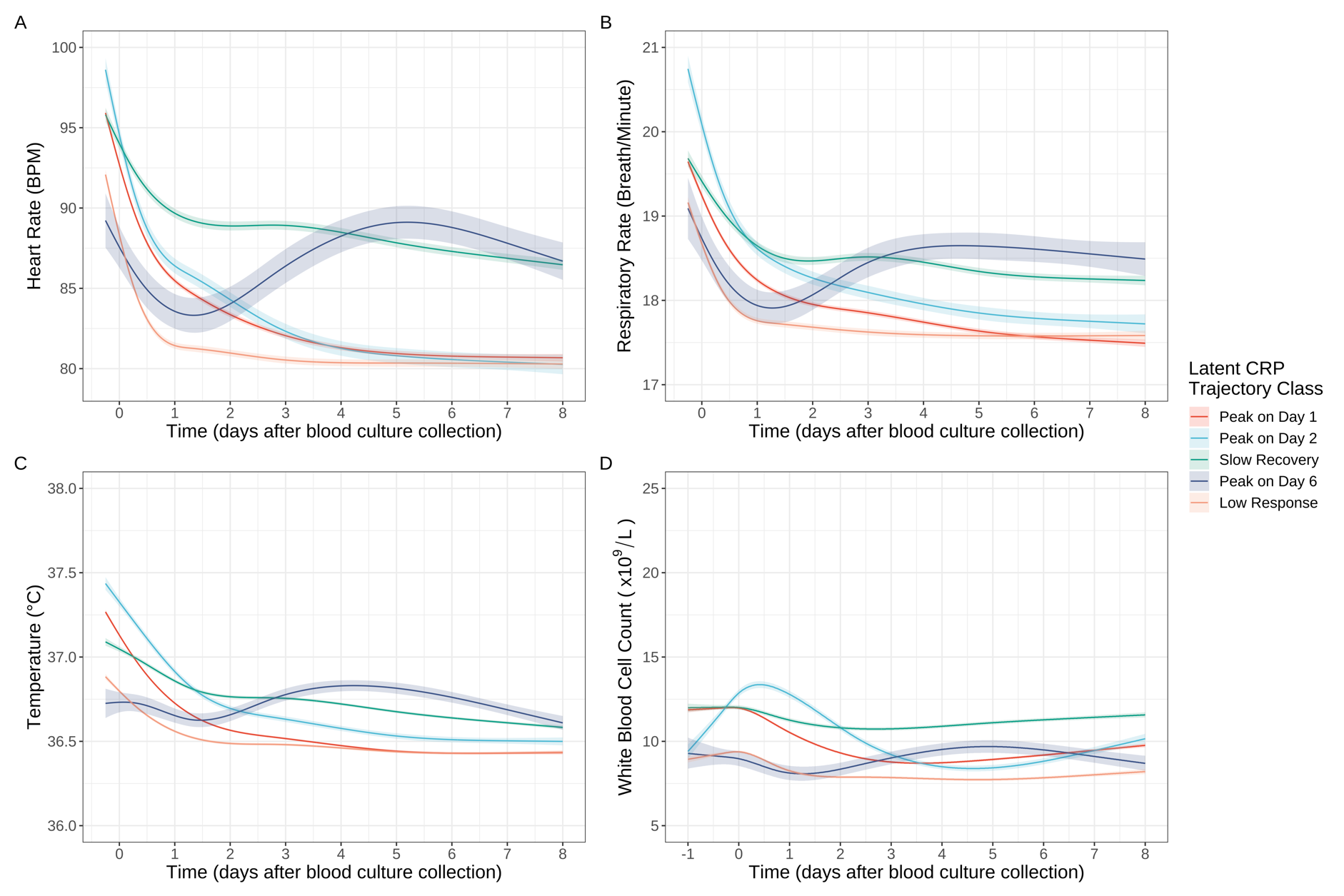


**Figure S10.** Response trajectories for heart rate (A), respiratory rate (B), temperature (C) and WBC count (D), by latent CRP trajectory class


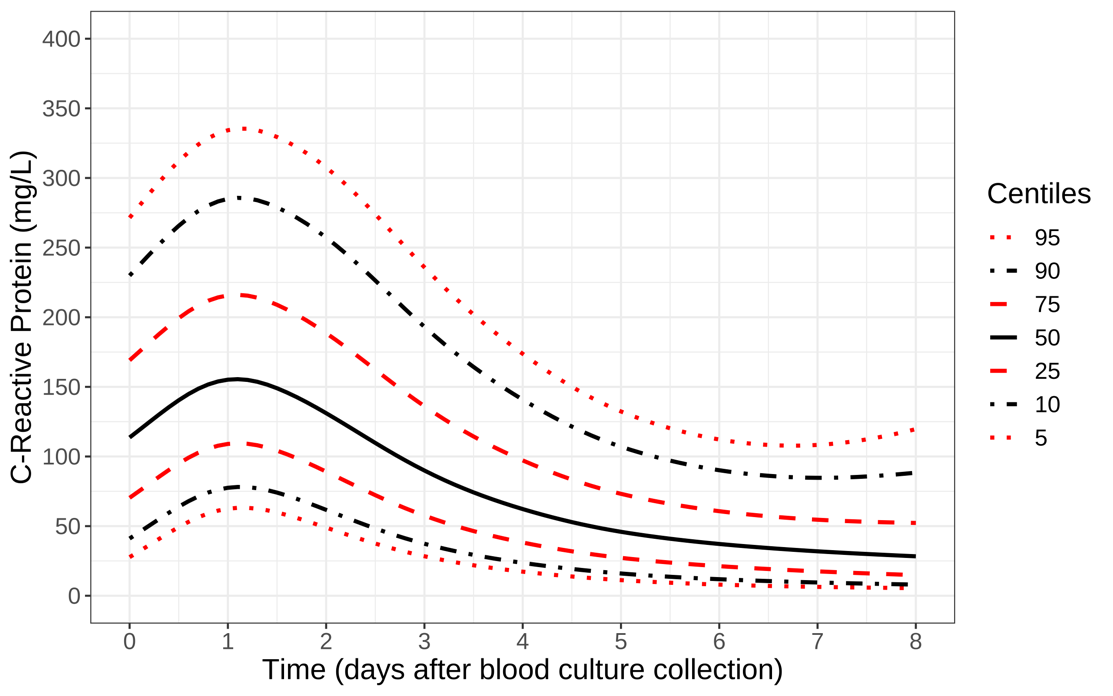


**Figure S11.** Centile reference chart estimated by selecting one random observation for each episode from those peaking on day 1 and day 2, regardless of pathogen isolated.


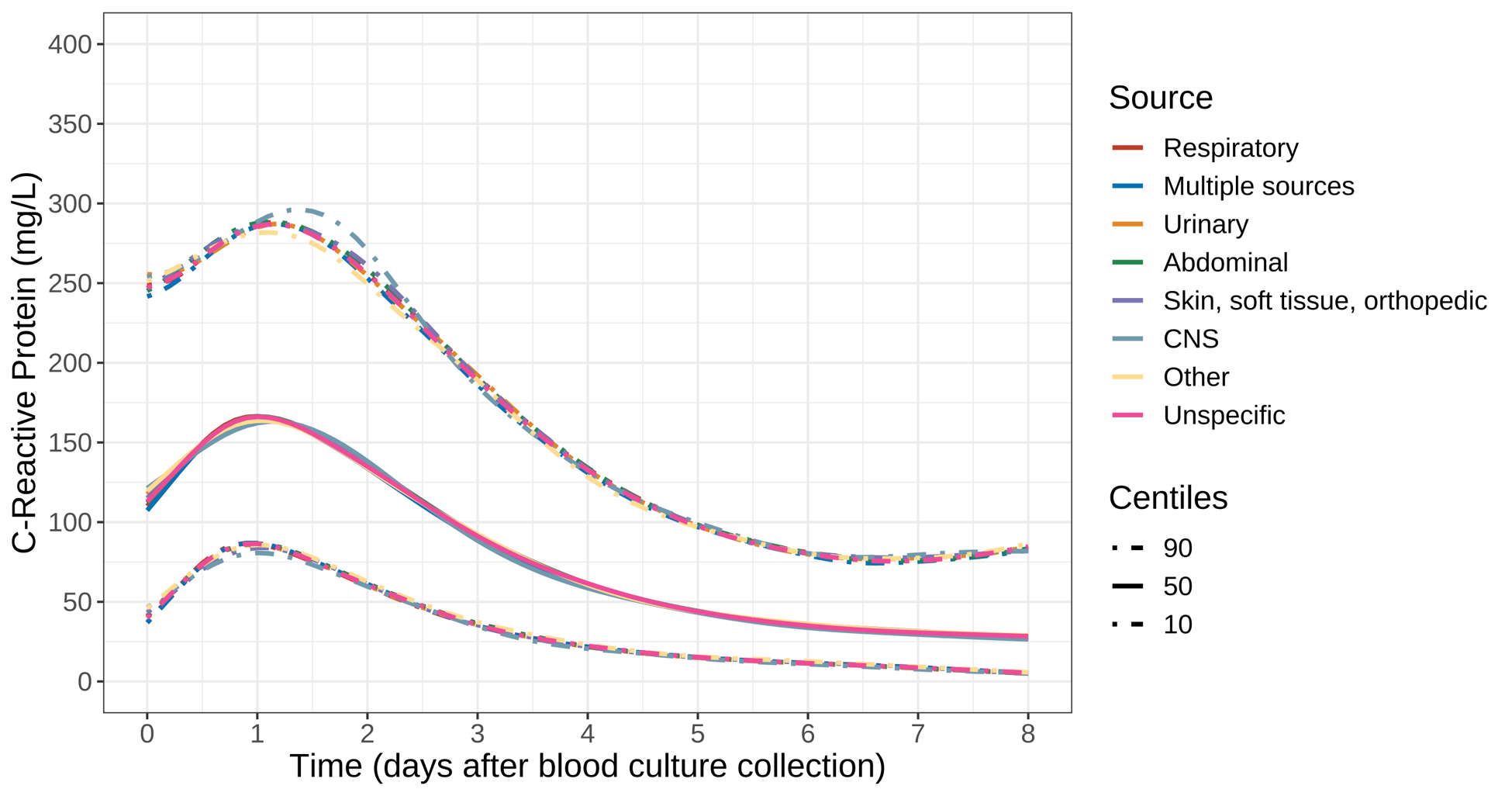


**Figure S12.** Centiles of expected CRP response in patients with culture-positive/negative suspected BSI following different sources of infection. Note: from the two latent classes peaking on day 1 and 2 in **Figure 2**, centiles for different sources of infection were estimated separately based on relevant episode subgroups, regardless of pathogen isolated.


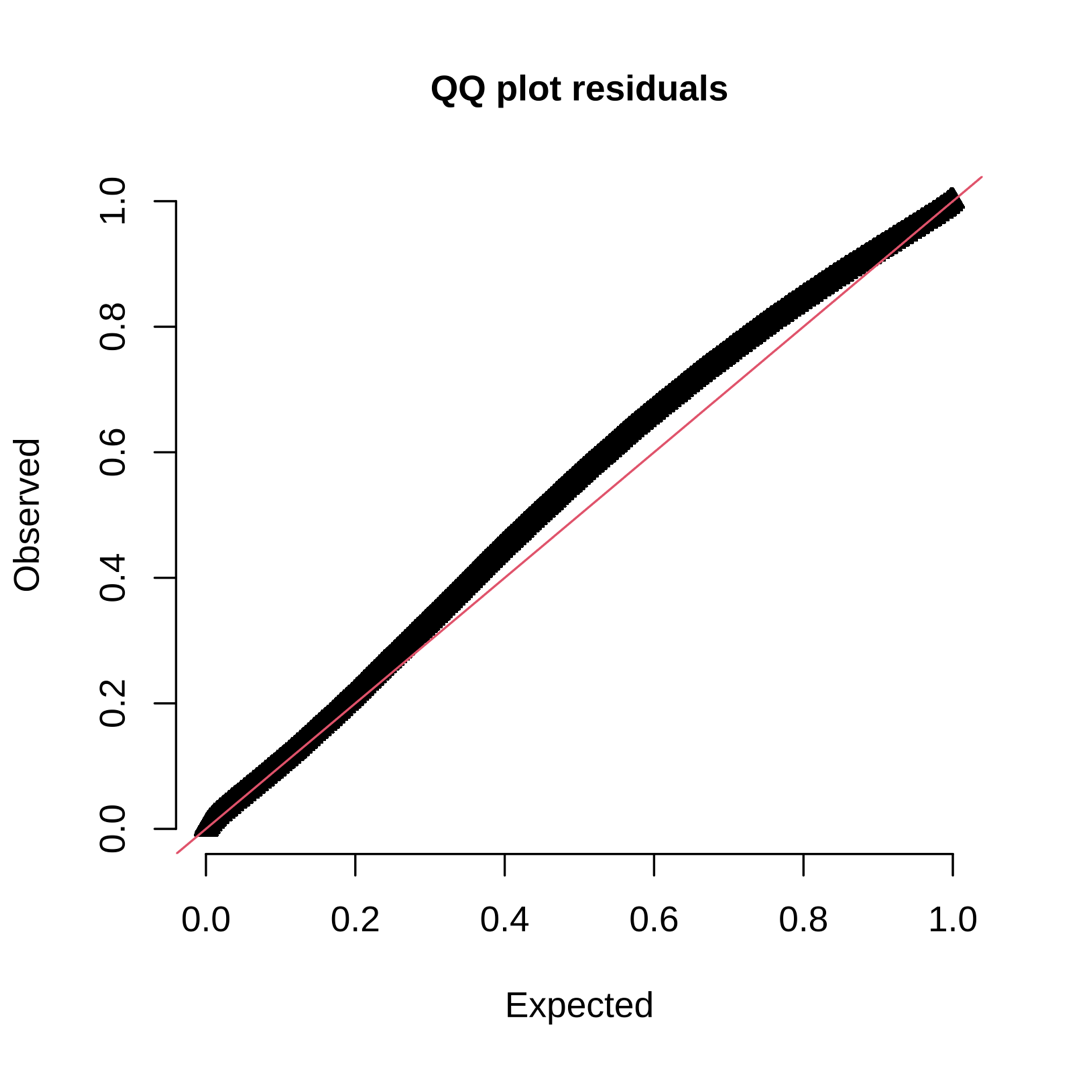


**Figure S13.** QQ plot of residuals for the main linear mixed model of CRP response trajectories.

# References

1 Pritchard Emma, Fawcett Nicola, Quan T. Phuong, Crook Derrick, Peto Tim EA., Walker A. Sarah. Combining Charlson and Elixhauser scores with varying lookback predicated mortality better than using individual scores. *J Clin Epidemiol* 2021;**130**:32–41. Doi: 10.1016/j.jclinepi.2020.09.020.

2 Yoon Chang Ho, Yuan Kevin, Gu Qingze, Munby Henry NP, Walker A. Sarah, Zhu Tinting, et al. Using Natural Language Processing on drug indications to predict working sources of infection. Machine Learning for Healthcare. Available at https://www.mlforhc.org. Accessed July 22, 2023, 2023.

3 Gilbert David N. *The Sanford Guide to Antimicrobial Therapy 2023*. Antimicrobial Therapy; 2023.

4 Weller Bridget E., Bowen Natasha K., Faubert Sarah J. Latent Class Analysis: A Guide to Best Practice. *J Black Psychol* 2020;**46**(4):287–311. Doi: 10.1177/0095798420930932.

5 Proust-Lima Cécile, Philipps Viviane, Liquet Benoit. Estimation of Extended Mixed Models Using Latent Classes and Latent Processes: The R Package lcmm. *J Stat Softw* 2017;**78**:1–56. Doi: 10.18637/jss.v078.i02.

6 Cole T. J., Green P. J. Smoothing reference centile curves: The lms method and penalized likelihood. *Stat Med* 1992;**11**(10):1305–19. Doi: 10.1002/sim.4780111005.

7 WHO Multicentre Growth Reference Study Group. WHO Child Growth Standards based on length/height, weight and age. *Acta Paediatr* 2006;**95**(S450):76–85. Doi: 10.1111/j.1651-2227.2006.tb02378.x.

8 Borghi E., de Onis M., Garza C., Van den Broeck J., Frongillo E. A., Grummer-Strawn L., et al. Construction of the World Health Organization child growth standards: selection of methods for attained growth curves. *Stat Med* 2006;**25**(2):247–65. Doi: 10.1002/sim.2227.

9 Rigby R. A., Stasinopoulos D. M. Generalized additive models for location, scale and shape. *J R Stat Soc Ser C Appl Stat* 2005;**54**(3):507–54. Doi: 10.1111/j.1467-9876.2005.00510.x.

10 Rigby Robert A, Stasinopoulos Dimitrios M. Automatic smoothing parameter selection in GAMLSS with an application to centile estimation. *Stat Methods Med Res* 2014;**23**(4):318–32. Doi: 10.1177/0962280212473302.

11 Wade Angela, Kurmanavicius Juozas. Creating unbiased cross-sectional covariate-related reference ranges from serial correlated measurements. *Biostatistics* 2009;**10**(1):147–54. Doi: 10.1093/biostatistics/kxn022.
